# Breast metastatic tumors in lung can be substituted by lung-derived malignant cells transformed by alternative splicing H19 lncRNA

**DOI:** 10.1186/s13058-023-01662-z

**Published:** 2023-05-30

**Authors:** Jin Biao Xu, Jun Cao, Jin Xia, Ying Zhu, Yi He, Ming Guo Cao, Bing Mu Fang, Jean Paul Thiery, Wu Zhou

**Affiliations:** 1 School of Medicine, Jiaxing University, Jiaxing, 314001 China; 2 University of Chinese Academy of Sciences, Chinese Academy of Sciences, Shanghai, 200031 China; 3 School of Medicine, Ningbo University, Ningbo, 3115211 China; 4 School of Medicine, Lishui University, Lishui, 323000 China; 5 Lishui City People’s Hospital, Lishui, 323000 China; 6 Guangzhou Laboratory, Guangzhou, 510700 China; 7 Institute of Molecular and Cell Biology, A-STAR, Singapore, 138673 Singapore

## Abstract

Metastasis accounts for most cancer-associated deaths; yet, this complex process remains poorly understood, particularly the relationship between distant metastasis and primary site-derived cells. Here, we modified the classical MMTV–PyMT breast carcinoma model to trace the fate of mammary-derived carcinoma cells. We show that within the lung, when the metastatic breast carcinoma cells are conditionally depleted, transformed lung epithelial cells generate new metastases. Metastatic breast carcinoma cells transmit H19 long noncoding (lnc) RNA to lung epithelial cells through exosomes. SF3B1 bearing mutations at arginine-625 alternatively splices H19 lncRNA in lung epithelial cells, which selectively acts like a molecular sponge to sequester let-7a and induces Myc upregulation. Under the conditional elimination of primary site-derived breast carcinoma cells, lung malignant cells expressing the mutated SF3B1 splice variant dominate the newly created tumors. Our study suggests that these new carcinoma cells originating from within the colonized organ can replace the primary site-derived malignant cells whenever their expansion is abrogated using an inducible diphtheria toxin receptor in our designed system. These findings should call for a better understanding of metastatic tumors with the specific origin during cancer metastasis.

**Supplementary Information:**

The online version contains supplementary material available at 10.1186/s13058-023-01662-z.

## Introduction

The overwhelming majority of cancer-associated deaths (about 90%) are caused by metastatic disease rather than primary tumors [1] and, despite significant advances in diagnosis and treatment, most patients with metastatic disease face a terminal illness that is, with rare exception, incurable with the current therapeutic regimens. Theoretically, the key to reducing rates of cancer mortality is to explore mechanisms driving cancer metastasis, with the expectation that such awareness would aid in the design of treatment strategies to eliminate cancer metastasis. However, this over-optimistic expectation is tempered by the unpredictable complexity of cancer metastasis. In spite of the wealth of knowledge pertaining to the detailed pathogenetic mechanisms of primary tumor formation, the multi-step process of metastasis remains the least understood aspect of cancer biology [2].

Metastasis has traditionally been considered a relatively late event within the linear progression model of tumorigenesis. A few recent reports, however, support the proposal of a parallel progression model [3], wherein dissemination can occur early during the process of neoplastic transformation, perhaps even before the departing cells are fully transformed [4, 5]. The main divergence between the linear and parallel progression models is whether early or late disseminated cells from the primary site are responsible for initiating metastasis in patients. For the propagation of metastasis, these two models consistently propose that the successful seeding of metastatic founder cells within distant tissues involves the formation of micrometastatic colonies in the parenchyma, followed by the proliferation of microscopic colonies into overt, clinically detectable metastatic lesions. Noteworthy, there is no comprehensive analysis as to how metastases pursue their development after the formation of metastatic lesions.

The continuous growth of metastatic tumors is considered to rely only on the expansion of the originally disseminated cells from the primary site—having further evolved in the distal organs—implying that therapeutics should target these cells. To some extent, this assumption does not account for the interaction between colonizing cancer cells and the secondary site microenvironment, which inevitably will in some part control any further metastatic development. The complex interplay among the host stromal environment, the primary tumor-derived factors, and the resident bone marrow-derived cells can prime and establish this ‘pre-metastatic niche’ [6]. Primary tumor-educated host stroma can serve to establish a supportive stromal microenvironment that may foster the colonization and outgrowth of the metastatic tumor cells in these secondary sites. The cellular and molecular components of the metastatic microenvironment, including the stromal cells, inflammatory cells, vascular networks, growth factors, nutrients, and other metabolic components, form a supportive ‘metastatic niche’ [7] to maintain the continuous growth of solid tumor metastasis. Stated differently, the metastases developing in the metastatic niche may promote on-site a deregulation of the targeted organ epithelium, as it is observed in the so-called field cancerization [8], and provide selective pressure for particular epithelial cells, which may, in turn, modify or even transform epithelial cells. To explore this possibility, we established transgenic mice based on the MMTV–PyMT mouse model of breast cancer to follow the fate of mammary and lung epithelial cells. Lung epithelial cells initially acquired an immortalized-like status and progressively expanded as malignant cells replacing breast metastatic carcinoma cells following their depletion by an inducible expression of diphtheria toxin receptors.

## Results

### Early dissemination of premalignant cells from mammary glands formed in MMTV–PyMT transgenic mice

**Fig. 1:**
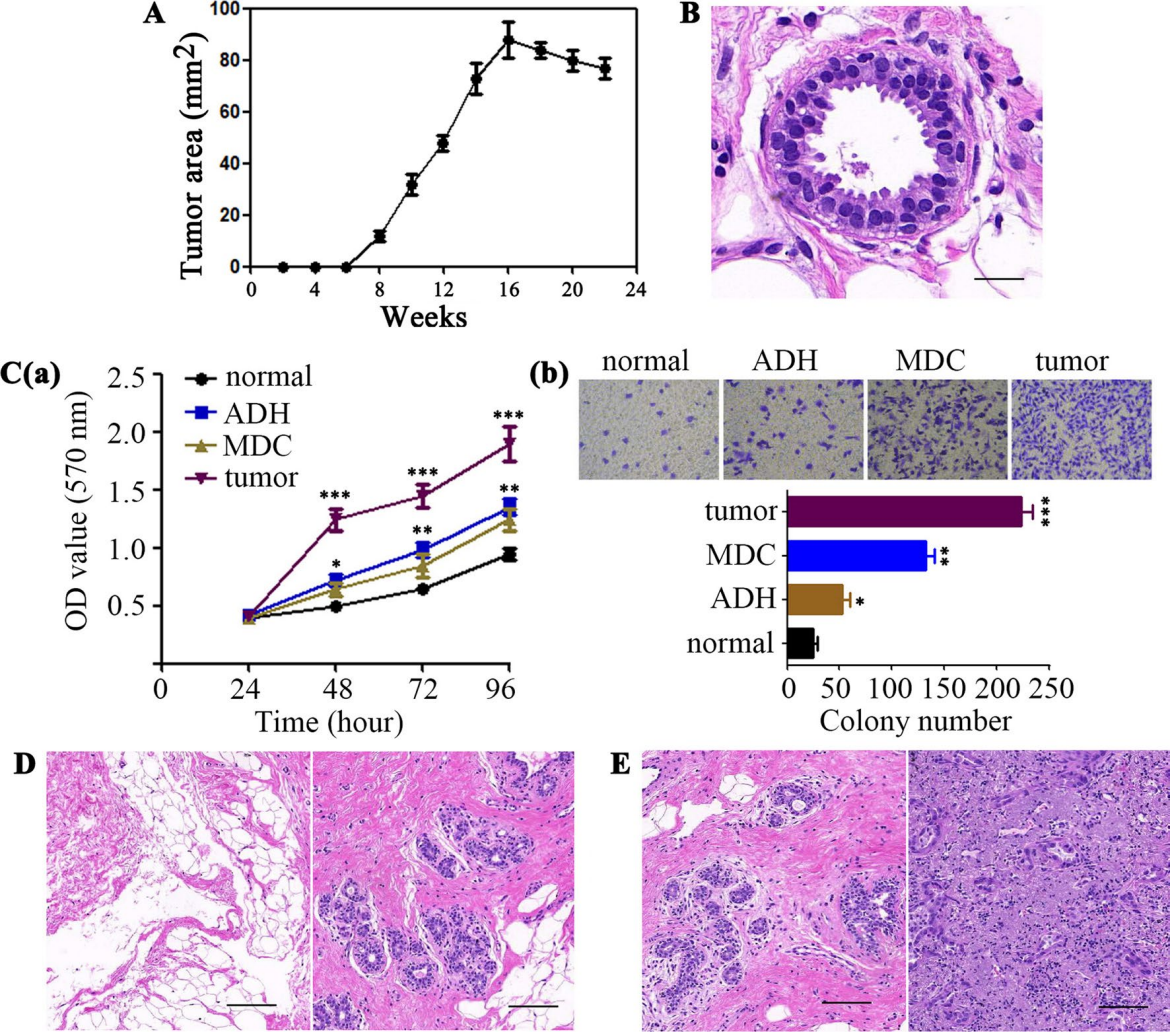
Early dissemination of mammary cells from the MMTV–PyMT transgenic model of breast cancer. **A** Increase in tumor area in MMTV–PyMT-Green (MPG) transgenic female mice (number of mice = 5) over time. Data are expressed as mean ± standard deviation. **B** The mammary gland and mammary gland-derived cells (MDCs) in MPG mice at 4 weeks (number of mice = 5). Histological sections of mammary glands showing that lateral buds display the morphology of atypical ductal hyperplasia (ADH). Scale bar, 50 μm. **C** Proliferation and invasive capacities in GFP-positive cells isolated from ADH areas (ADH) or lung tissues (MDC) of 4-week-old MPG mice, normal cells (normal) digested from wild-type syngeneic mice and GFP-positive cells isolated from the breast tumor (tumor) of 12-week-old MPG mice, as assessed by MTT (**a**) and in vitro invasion assay (**b**), respectively. Data were quantified and presented as the mean ± SD of triplicate experiments. *P < 0.05, **P < 0.01, ***P < 0.001by Student’s *t-*test. **D** GFP-positive cells isolated from ADH areas of 4-week-old MPG mice (donor mice = 8) were transplanted subcutaneously (left panel) or orthotopically (right panel) into syngeneic wild-type (WT) recipient mice (1 million cells/mice, recipient mice = 40). Histopathology of transplanted mammary cells at 18 weeks after transplantation. Scale bar, 200 μm. **E** FACS-sorted early MDC cells (E-MDCs) from the lung of MPG mice (donor mice = 20) at 4 weeks were transplanted into the mammary gland of syngeneic WT recipient animals (1 million cells/mice, recipient mice = 20). Histopathology of the mammary gland at 4 weeks (left panel) or 18 weeks (right panel) after transplantation. *n* = number of donor or recipient mice. Scale bar, 200 μm

To better understand the relationship between distant metastasis and primary site-derived cells, we used the transgenic mammary tumor virus–polyomavirus middle T antigen (MMTV–PyMT) mouse model, which reproduces the stepwise progression of breast cancer with high metastatic dissemination to the lungs [9]. For tracing mammary gland-derived cells, we generated a genetic lineage MMTV–PyMT-Green (MPG) mouse that uses the Cre-loxP recombination system to label mammary gland-derived epithelial cells with GFP (Additional file 1: Fig. 1A). In MPG transgenic female mice, progression to in situ carcinoma occurs between 5 and 8 weeks of age. Around week 8, tumors of the mammary glands become palpable or visible and, at 12–15 weeks, invasive carcinoma becomes apparent (Fig. 1A).

At the early age of 4 weeks, only the areas of atypical ductal hyperplasia (ADH) were measured in MPG mice (Fig. 1B). Compared with normal cells, fluorescent cells isolated from ADH areas of 4-week-old transgenic mammary glands had no stronger invasion ability in vitro, but the proliferation ability of cells was enhanced (Fig. 1C). These cells were further transplanted both subcutaneously (ectopic) and into gland-cleared fat pads (orthotopic) of syngeneic wild-type (WT) recipient mice. The results showed that none of the sorting cells transplanted subcutaneously (0 of 20) or orthotopically (0 of 20) formed palpable tumors within 12–18 weeks post-transplantation (Additional file 1: Fig. 1B). However, whole-mount analysis of the mammary glands revealed that although there was no histological change in the subcutaneous transplanted mice, there was noninvasive hyperplasia in the fat pad transplanted mice (Fig. 1D). These data suggest that, at 4 weeks, the fluorescent epithelial cells of the mammary gland have not yet acquired a malignant phenotype.

Moreover, the malignant potential was also investigated in early disseminated cells. Our results showed that, in MPG mice, mammary gland-derived cells were detectable in the bone marrow and lung at as early as 4 weeks of age (Additional file 1: Fig. S1C). In the early stage, the proliferation ability of cells disseminated in the lung was not much changed than that of ADH cells in vitro, but the invasion ability of cells increased (Fig. 1C). Besides, these cells were subcutaneously transplanted into syngeneic WT recipient animals. These ectopic (s.c.) transplants did not progress to tumors (0 of 20), providing strong evidence they were not malignant. However, in orthotopically transplanted mice, most of the early disseminated cells (17 of 20) gave rise to ADH areas and eventually developed tumors within the recipient fat pad, indicating that the early disseminated cells from breast ADH tissues are premalignant cells (Fig. 1E and Additional file 1:Fig. 1B). These early mammary gland-derived cells (E-MDCs) had a high incidence of malignant transformation and grew into tumors in orthotopic sites, confirming their potential for malignant progression.

### Resident cells are involved in the continuous growth of distant metastases

To further illustrate the role of MDCs in cancer metastasis, we assessed the development of lung metastasis in MPG mice. As expected, lung micrometastases were found at 12 weeks of age. Older mice (16-week-old) showed metastatic foci lodged within the lung parenchyma. The GFP-positive cells isolated from these micrometastases or metastatic tumors were subcutaneously or orthotopically transplanted into syngeneic WT recipient animals. The results showed that all recipient mice formed tumors within 18 weeks (Additional file 1: Fig. S1B), demonstrating the malignant transformation of MDCs. Furthermore, we measured the lung of MMTV-Green mice (#3 in Additional file 1: Fig. S1B) and MPG mice (#5 in Additional file 1: Fig. S1A) and found that no GFP fluorescent cells were detected in the lung of MMTV-Green mice, excluding the possibility that the GFP-positive malignant cells were induced by MMTV of resident cells inside the lung (Additional file 2: Fig. S2).

**Fig. 2:**
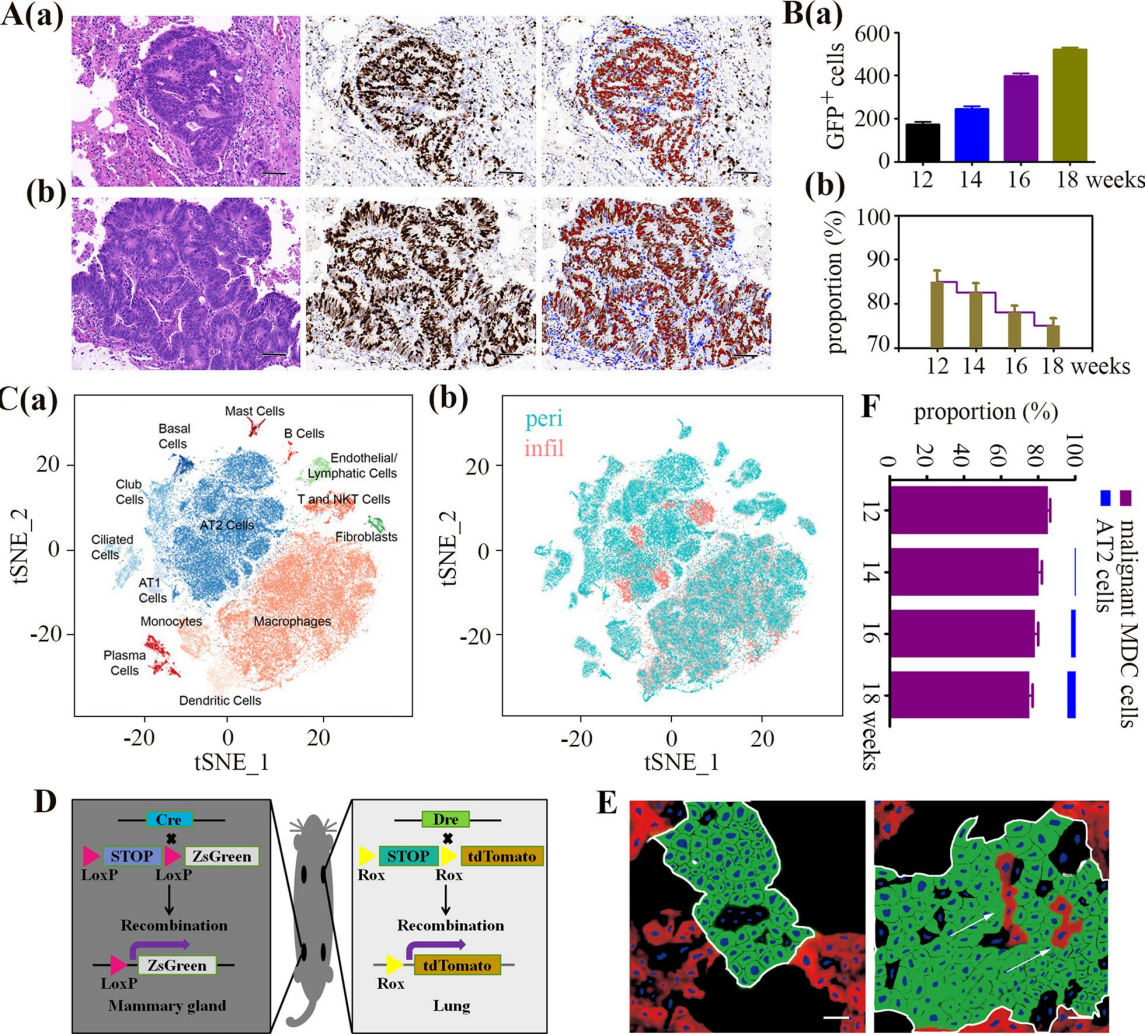
Metastasis expansion is not dependent on malignant MDCs. **A** H&E staining (left panel) or GFP immunohistochemistry (middle panel) of lung metastases forming in MMTV–PyMT-Green (MPG) mice at 12 (**a**) or 16 weeks (**b**). Blue, DAPI staining. To quantify the proportion of GFP-positive cells (right panel), the layers of images were decomposed by Image J software and restained using chromophore fluorescence for GFP expression (red) and DAPI (blue). Scale bar, 50 μm. **B** The absolute numbers (**a**) and proportion (**b**, number of GFP-positive cells divided by the total number of cells) of malignant mammary gland-derived cells (MDCs) in lung metastases of MPG mice (7 metastases/group, *n* = 5) at different ages (from 12 to 18 weeks) was quantified. *n* = number of groups. Data are expressed as mean ± standard deviation. **C** Single-cell RNA-Seq was performed on single-cell suspensions generated from the infiltrating cells (excluding T lymphocytes) or peritumoral cells of the lung metastatic tissues of 16-week-old MPG mice. **a** Cells were clustered using a graph-based shared nearest neighbor clustering approach and visualized using a t-distributed Stochastic Neighbor Embedding (tSNE) plot. **b** Cells on the tSNE plot were colored as originating either from the infiltrating cells (Infil) or peritumoral cells (Peri). **D** Schema of the genesis of MMTV–PyMT-Green/Sftpc-Tomato mice (MPG-ST) mice. **E** Reconstructed confocal images of the lung sections collected from MPG-ST mice at 12 (left panel) and 16 (right panel) weeks. The white frames showed metastases, and the arrows showed interspersed red fluorescent cells. Blue, DAPI; Green, malignant MDCs; Red, AT2 cells; Scale bar, 20 μm. **F** The proportion of malignant MDCs and AT2 cells in lung metastases of MPG-ST mice at different ages was quantified. Data are expressed as mean ± standard deviation

Surprisingly, on closer inspection, we found that the proportion of malignant MDCs (M-MDCs) in the micrometastases was not consistent with that in the macrometastases (Fig. 2A). Histological examination of MPG mice revealed that, with the progression of metastasis, there was a significant drop in the percentage of M-MDCs (Fig. 2B). This observation indicates that an increasing number of non-fluorescent cells were recruited into the metastatic foci during the progression of metastasis. To identify the origin of these infiltrating cells, we performed Single-cell RNA-Seq on the lung metastasis infiltrating cells and cells isolated from peritumoral tissue of lung metastasis of 16 weeks old MPG mice. We assigned cell types to each cluster based on the expression of established markers from the LungMAP databases [10]. In the cells from the peritumoral tissue of lung metastasis, we identified alveolar type II cells (AT2 cells), alveolar type I cells (AT1 cells), ciliated, club, and basal airway epithelial cells, macrophages, dendritic cells, T cells and other cells (Fig. 2C(a)), while the infiltrating cells were mainly composed of immune cells, and AT2 cells (Fig. 2C(b)).

To verify the involvement of AT2 cells in lung metastatic tumors, we established a Dre transgenic mouse model using AT2 cell marker surfactant protein C (Sftpc) [11] (Additional file 3: Fig. S3). This mouse was crossed with an RSR reporter mouse [12] to produce Sftpc-tdTomato mice (ST mice), where Sftpc-positive cells were labeled with tdTomato. The transgenic ST mice were then crossed with MPG mice to obtain MMTV–PyMT-Green/Sftpc-Tomato mice (MPG-ST mice). Under these two recombination systems, Cre-loxP and Dre-rox (Fig. 2D), MPG-ST mice lung AT2 cells were individually labeled with red fluorescence in addition to the characteristics of MPG mice (Additional file 4: Fig. S4). Lung micrometastases could be detected in 12-week-old MPG-ST and MPG mice, yet no red fluorescence was observed at this time. However, in older mice (16-week-old), lung macrometastatic foci were interspersed with red fluorescent cells (Fig. 2E). These results suggest that an increasing number of AT2-derived cells were incorporated into the growing secondary tumors (Fig. 2F).

### Newly generated growth of distant metastasis is not dependent on malignant MDCs

**Fig. 3:**
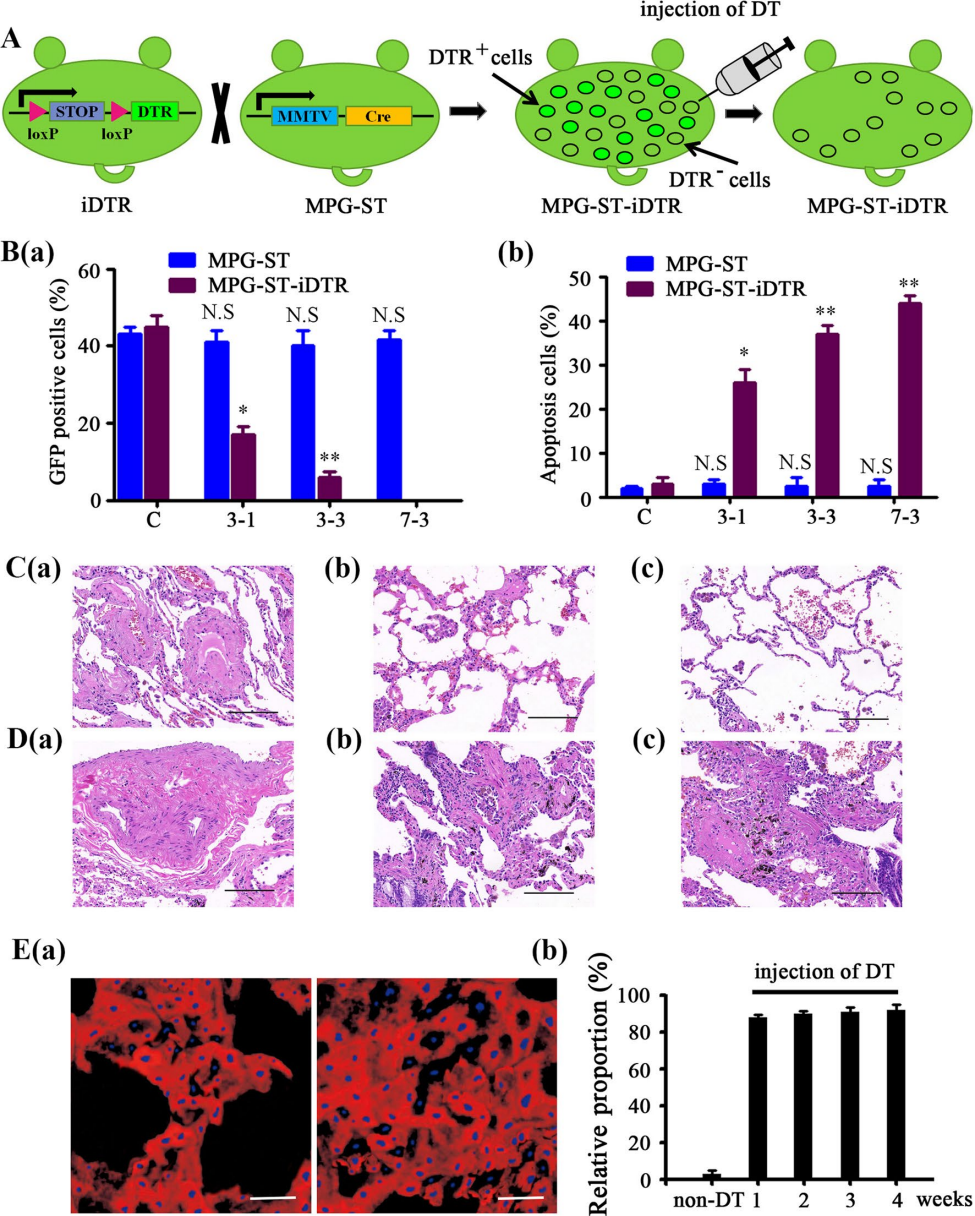
The newly generated growth of distant metastasis is not dependent on malignant MDCs. **A** Scheme of the Cre-inducible diphtheria toxin receptor (DTR) transgenic mice (iDTR) system. Crossing the iDTR strain with the MMTV–PyMT-Green/ Sftpc-tdTomato (MPG-ST) strain renders Cre-induced GFP-positive cells sensitive to DT. **B** Four-week-old MPG-ST and MPG-ST-iDTR mice (n = 6) were injected with 100 ng DT once daily for 3 consecutive days (3-1), 3 times a day for 3 consecutive days (3-3), or 3 times a day for 7 consecutive days (7-3). Mammary glands were analyzed 36 h later. The percentage of GFP-positive cells (**a**) or apoptotic cells stained with Annexin V (**b**) in different conditions are indicated. Mice without treatment by DT were used as control (**C**). Data are Mean ± s.d., *n* = 3 independent experiments per condition. N.S, no significance; *P < 0.05, **P < 0.01 by Student’s *t* test. MPG-ST-iDTR mice at 12 weeks (**C**) or 16 weeks (**D**) were injected with 100 ng diphtheria toxin (DT) every 8 h for 7 consecutive days. Lung metastases were detected by H&E staining (**a**–**c**; Scale bar, 200 μm). Representative images of lung sections without DT treatment (**a**) and at 1 week (**b**) or 2 weeks (**c**) after DT injection. **E** At 16 weeks, MPG-ST-iDTR mice were injected with 100 ng DT every 8 h for 7 consecutive days. **a** Reconstructed confocal images of the lung sections were taken at 1 week (left panel) or 2 weeks (right panel) after DT injection. Blue, DAPI; Red, AT2 cells; Scale bar, 50 μm. **b** The proportion of AT2 cells in lung metastases of MPG-ST-iDTR mice before (non-DT) or after DT treatment. All experiments were performed at least 3 times. Data are expressed as mean ± standard deviation. Abbreviations: MPG, MMTV–PyMT-Green; MGP-ST, MPG/Sftpc-Tomato; MPG-ST-iDTR, MGP/ST/Cre-inducible diphtheria toxin receptor

Given that the proportion of AT2-derived cells in metastatic lesions gradually increased yet that of M-MDCs declined, we sought to determine how metastases could evolve with a decreasing number of M-MDCs until their complete disappearance. To address this point more directly, we crossed MPG-ST mice with Cre-inducible diphtheria toxin receptor (DTR) transgenic mice (iDTR) [13], in which the Cre-mediated excision of a STOP cassette renders cells sensitive to diphtheria toxin (DT). We first tested whether the Cre-inducing iDTR system functions in combination with the Cre-expressing MPG-ST mouse strain (Fig. 3A) and found that the GFP-positive cells in the mammary glands of MPG-ST-iDTR mice can be selectively depleted upon the injection of DT (Fig. 3B). Thus, deletion of the STOP cassette in MDCs confers their sensitivity to DT-mediated cell death.

The requirement of M-MDCs for continuous metastatic growth was thus evaluated by selective depletion experiments. We show that the most stringent application of DT into MPG-ST-iDTR mice could result in a complete clearance of M-MDCs in lung and therefore the regression of metastases (Fig. 3C and Additional file 5: Table S1). However, this phenomenon was only observed in MPG-ST-iDTR mice under 15 weeks of age, which are within the early stage of metastasis. In MPG-ST-iDTR mice older than 15 weeks, which have already developed pulmonary macrometastases, the elimination of MDCs via DT injection failed to prevent the subsequent growth of metastatic tumors. After DT treatment, the metastatic tumors transiently reduced in size but then re-grew afterward (Fig. 3D and Additional file 5: Table S1). It is worth noting that after DT treatment, AT2-derived cells expressing Sftpc protein increased rapidly and turned into the main component in lung metastases without M-MDCs (Fig. 3E and Additional file 6: Fig. S5). These findings demonstrate that M-MDCs are dispensable for the continuous growth of metastatic tumors. Alveolar epithelial cells participate in the development of lung metastases, and in the absence of M-MDCs, these cells dominate the newly generated growth of metastases.

### Malignant MDCs transfer H19 lncRNA through secreting exosomes

**Fig. 4:**
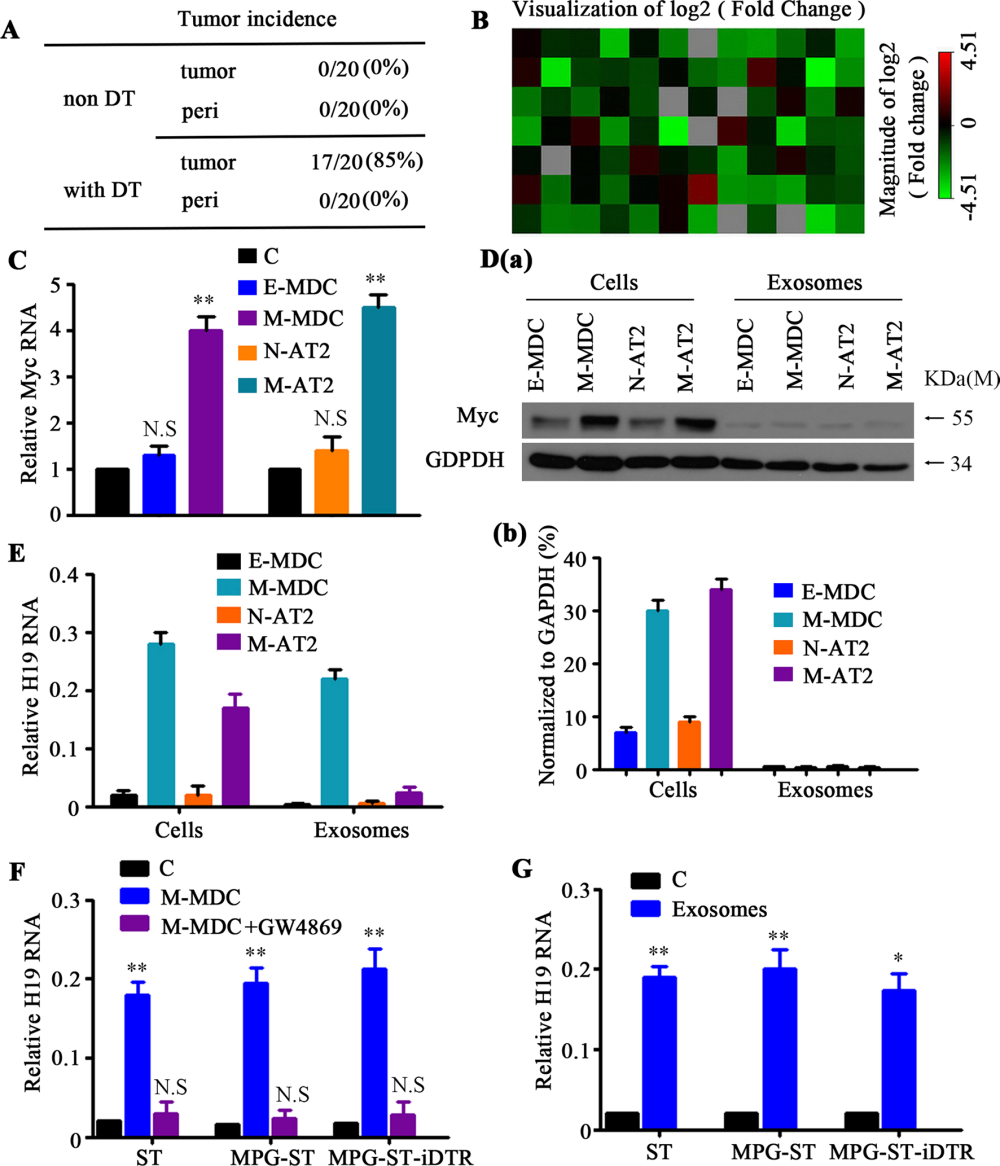
Malignant MDCs transfer H19 LncRNA through secreting exosomes. **A** Sftpc-expressing AT2 cells sorted from lung metastatic tumors (tumor) or their peritumoral tissues (peri) of 16-week-old MPG-ST-iDTR mice with or without DT (non-DT) injection were subcutaneously transplanted into wild-type (WT) recipient mice. Tumor incidence, described as transplantation sites with tumors/transplanted mice amount (ratio), was measured at 18 weeks after transplantation. **B** PCR arrays were used to compare the expression of oncogenes and tumor suppressor genes between malignant AT2 and non-malignant AT2 groups. The red and green colors represent, respectively, increased or decreased gene expression in the heatmap for the malignant and non-malignant AT2 groups. Myc located at F07 (Extended Data Fig. 6A). **C** qRT-PCR analysis for Myc expression in the indicated cells. E-MDC and M-MDC cells were GFP-positive cells FACS sorted from the lungs of 4-week-old (E-MDC, *n* = 15) or 16-week-old (M-MDC, *n* = 3) MPG-ST-iDTR mice (GFP-positive cells FACS sorted from the mammary ADH tissue of 4-week-old MPG-ST-iDTR mice were used as control); N-AT2 and M-AT2 cells were tdTomato-positive cells FACS sorted from the parenchyma (N-AT2, *n* = 3) or lung metastasis (M-AT2, *n* = 3) of the MPG-ST-iDTR mice 2 weeks after DT treatment (tdTomato-positive cells FACS sorted from the peritumoral tissues of MPG-ST-iDTR mice before DT treatment were used as the control). *n* = number of mice. Data presented are the mean of triplicate experiments. Error bars indicate standard deviation (**P < 0.01; N.S, No significance). **D** Immunoblotting for anti-Myc in E-MDCs, M-MDCs, N-AT2 cells, M-AT2 cells (as described in **C** and their secreted exosomes (**a**). Quantification of Myc expression level (**b**). GAPDH was used as a control. Data presented are the mean of triplicate experiments. Error bars indicate standard deviation. **E** RNA expression of H19 lncRNA in the indicated cells (as described in **C**) and their secreted exosomes were analyzed by qRT-PCR and normalized to U6 snRNA. Data presented are the mean of triplicate experiments. Error bars indicate standard deviation. **F** and **G** FACS-sorted tdTomato-positive cells from the lung of 4-week-old ST mice, MPG-ST mice, or MPG-ST-iDTR mice were co-cultured with M-MDCs (as described in** C**) or M-MDCs with exosome inhibitor GW4869 **(F**, without M-MDCs as control) or with just exosomes collected from M-MDCs (**G**, without exosomes as control). The RNA level of H19 LncRNA was measured by qRT-PCR and normalized to U6 snRNA. Data presented are the mean of triplicate experiments. Error bars indicate standard deviation (**P < 0.01; *P < 0.05). Abbreviations: MPG, MMTV–PyMT-Green; MGP-ST, MPG/Sftpc-Tomato; MPG-ST-iDTR, MGP/ST/Cre-inducible diphtheria toxin receptor; MDCs, mammary gland-derived cells; E-MDCs, early MDCs; M-MDCs, malignant MDCs; N-AT2, non-malignant AT2; M-AT2, malignant AT2; C, control

Because metastases continued to grow in the absence of M-MDCs, we sought to determine if the dominating AT2 cells had acquired a malignant phenotype. We therefore isolated AT2-derived cells from metastatic tumors and their non-malignant adjacent tissues from a 16-week-old MPG-ST-iDTR mouse having been subjected to DT treatment and transplanted these cells into syngeneic WT recipient animals. We found that AT2 cells localized in the metastatic tumors developed into tumors but not those cells located in the adjacent parenchyma (Fig. 4A). In the absence of M-MDCs, AT2 cells formed the bulk of the metastatic tumor, which have acquired autonomously a malignant phenotype confirmed by lung adenocarcinoma marker Napsin A and TTF-1 (Additional file 7: Fig. S6). These data demonstrated that the normal pulmonary AT2 cells inside the lung metastatic tumors were transformed, leading us to ask how the normal AT2 cells acquire the malignant phenotypes?

The major alterations that can lead to malignant transformation of cells fall into two categories: (1) gain-of-function mutations in proto-oncogenes, which stimulate cell growth, division, and survival; and (2) loss-of-function mutations in tumor suppressor genes, which would normally help to prevent unrestrained cellular growth and promote DNA repair and cell cycle checkpoint activation [14]. To explore whether proto-oncogenes or tumor suppressor genes were implicated in the malignant transformation of pulmonary AT2 cells, we used a combined oncogene and tumor suppressor gene PCR array. We analyzed and compared the expression of 84 core genes (Additional file 8: Data file S1) in lung metastatic AT2 (malignant AT2, M-AT2) cells and the parenchyma non-malignant AT2 (N-AT2) cells from MPG-ST-iDTR mice after DT treatment. We found high expression of the proto-oncogene Myc in M-AT2 cells (Fig. 4B). These results were validated by qRT-PCR experiments, which showed higher expression of Myc in M-MDCs in lung metastasis as compared with E-MDCs (Fig. 4C). Overall, these data suggest that M-MDCs and M-AT2 cells were transformed by Myc overexpression.

Myc expression was not abnormal in N-AT2 cells but was upregulated in malignant M-MDCs and infiltrating M-AT2 cells of lung metastasis (Fig. 4C). Thus, we speculated that Myc may be transmitted between M-MDCs and M-AT2 cells. Exosomes usually transfer DNA, RNA, lipids, metabolites, and cytosolic and cell surface proteins from cancer cells to their surrounding cells [15]. We firstly detected the possibility of Myc passing from M-MDC to infiltrating AT2 cells around them through exosomes. Exosomes were extracted and purified from E-MDC, M-MDC, N-AT2 cells, and M-AT2 cells by ultracentrifugation. Released exosomes were characterized by morphology under transmission electron microscopy (Additional file 9: Fig. S7A), as well as via the expression of classical (e.g., CD9, CD63, CD81) biomarkers (Additional file 9: Fig. S7B). Unexpectedly, although Myc protein expression levels in M-MDC or M-AT2 cells were higher than those in E-MDC or N-AT2 cells, high level of Myc was not detected in their exosomes (Fig. 4D), indicating that Myc was not transmitted between the two types of cells through exosomes.

Thus, we then asked whether long noncoding RNA (lncRNA) may regulate Myc for transformation through exosomes between M-MDCs and AT2 cells. The high throughput sequencing was conducted to explore the lncRNA expression profile of the exosomes from E-MDC and M-MDC cells. As shown in Additional file 10: Data file S2, 1199 known lncRNAs were expressed in each exosome. We compared the lncRNA expression levels between the exosomes from E-MDC and M-MDC cells and identified 20 long ncRNAs that were highly expressed in the exosomes from M-MDC cells (≥ twofold and FDR ≤ 0.001) (Additional file 11: Table S2). We next focused on H19 lncRNA, which was known to act as a selective molecular sponge in the sequestration of Let-7b [16], and others have reported that Let-7a downregulates Myc upregulation [17]. The RNA expression of H19 lncRNA in E-MDCs, M-MDCs, M-AT2 cells, N-AT2 cells, and their secreted exosomes, was analyzed by qRT-PCR. H19 lncRNA was highly expressed in M-MDCs and M-AT2 cells over that in E-MDCs and N-AT2 cells (Fig. 4E). However, the expression of P53 was downregulated in M-MDCs, comparing with that in E-MDCs, N-AT2 and M-AT2 cells (Additional file 9: Fig. S7C). Since P53 can normally suppress the expression of H19 lncRNA [18] and there was a significant increase in H19 lncRNA in exosomes secreted by M-MDCs (Fig. 4E), we infer that H19 lncRNA was promoted by different mechanism between M-MDCs and M-AT2 cells, and M-MDCs may transmit H19 lncRNA to AT2 cells through exosomes.

To verify this hypothesis, normal AT2 cells from 4-week-old ST mice, MPG-ST mice, or MPG-ST-iDTR mice were co-cultured with M-MDCs and then sorted for H19 expression. We found that H19 dramatically increased in normal AT2 cells after being co-cultured with M-MDCs and noted that this increase could be blocked by the exosome inhibitor GW 4869 (Fig. 4F). More directly, we collected exosomes of M-MDCs with a high expression of H19 and co-cultured them with normal AT2 cells from 4-week-old ST, MPG-ST, or MPG-ST-iDTR mice. We found a higher expression of H19 in these normal AT2 recipient cells (Fig. 4G) as compared with control. Together, these results demonstrate that M-MDCs secrete exosomes harboring H19 lncRNAs and transmit them to lung alveolar AT2 cells.

### H19 LncRNA induces the immortalization of the recipient cells

**Fig. 5:**
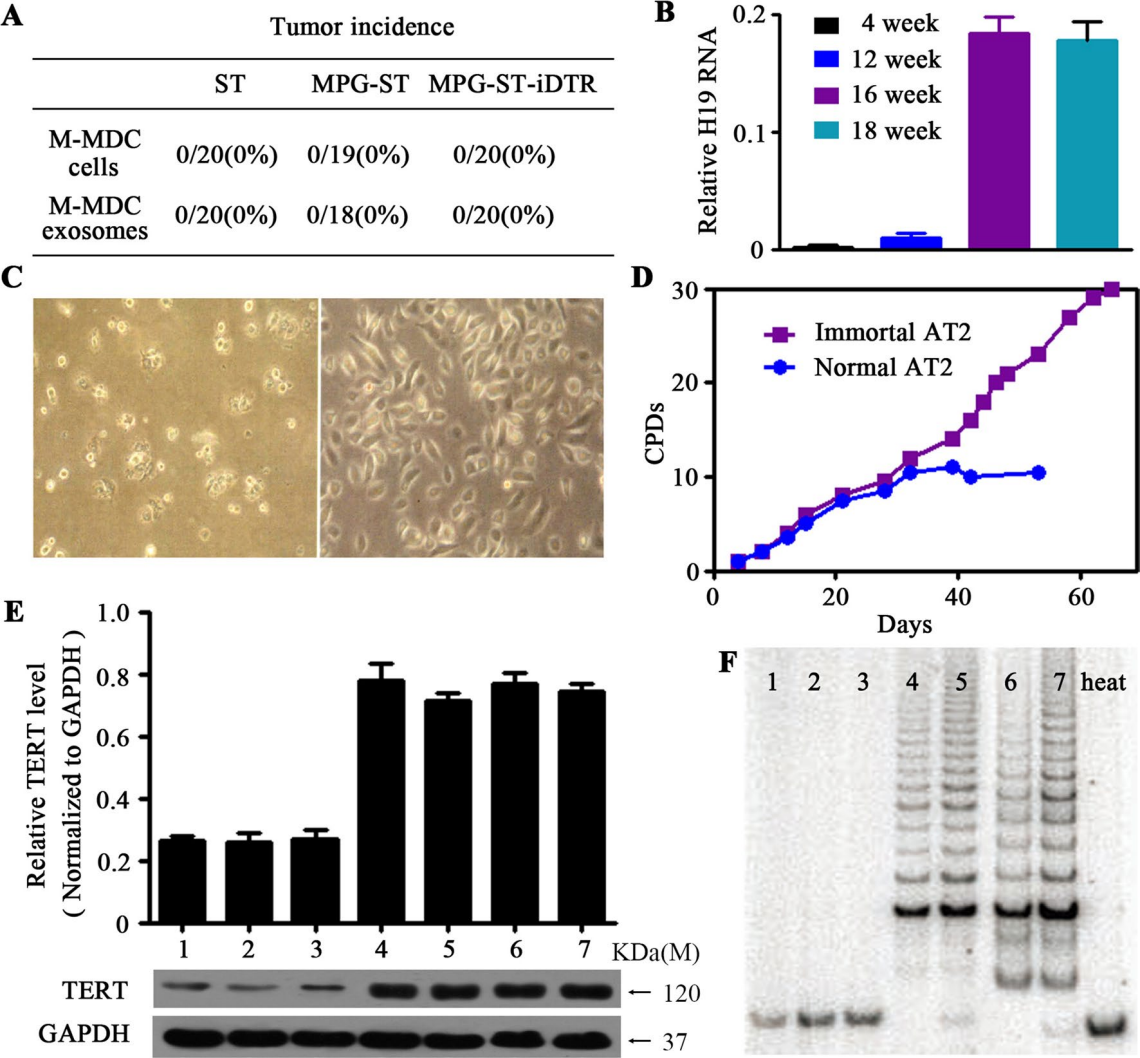
H19 lncRNA induces the immortalization of recipient cells. **A** FACS-sorted tdTomato-positive cells from the lung (normal AT2 cells) of 4-week-old ST mice (*n* = 20), MPG-ST mice (*n* = 20), or MPG-ST-iDTR mice (*n* = 20) were co-cultured with M-MDCs (as described before) or their exosomes and then transplanted into wild-type (WT) recipient mice. Tumor incidence, described as transplantation sites with tumors/transplanted mice amount (ratio), was measured at 18 weeks after transplantation. *n* = number of mice. **B** The tdTomato-positive AT2 cells were sorted from the lung metastasis of MPG-ST-iDTR mice (without DT injection) at 12 weeks, 14 weeks, 16 weeks or 18 weeks (*n* = 5/group). The RNA levels of H19 lncRNA were determined by qRT-PCR and normalized to U6 snRNA. *n* = number of mice. Data presented are the mean of triplicate experiments. Error bars indicate standard deviation. **C** Morphology of 6th passage primary AT2 cells from 12 to week-old MPG-ST-iDTR mice (left panel) or 10th passage primary AT2 cells from lung metastases of 18-week-old MPG-ST-iDTR mice (right panel). **D** Cumulative population doublings (CPDs) show the growth kinetics of the primary AT2 cells from 12-week-old MPG-ST-iDTR mice (normal AT2) or primary AT2 cells from lung metastases of 18-week-old MPG-ST-iDTR mice (immortal AT2). **E** Expression of telomerase reverse transcriptase (TERT) in samples described below was detected and quantified. GAPDH was used as a control. Data presented are the mean of triplicate experiments. Error bars indicate standard deviation. **F** Telomerase activity of samples described below was determined by telomeric repeat amplification protocol assay. As a control, heat-inactivated samples were also assayed (right lane). Samples of **E** and **F** lane 1: normal AT2 cells from ST mice; Lane 2: normal AT2 cells from MPG-ST mice; Lane 3: normal AT2 cells from MPG-ST-iDTR mice; Lane 4: normal AT2 cells from ST mice co-cultured with exosomes from M-MDCs; Lane 5: normal AT2 cells from MPG-ST mice co-cultured with exosomes from M-MDCs; Lane 6: normal AT2 cells from MPG-ST-iDTR mice co-cultured with exosomes from M-MDCs; Lane 7: immortal AT2 cells. Abbreviations: MPG, MMTV–PyMT-Green; MGP-ST, MPG/Sftpc-Tomato; MPG-ST-iDTR, MGP/ST/Cre-inducible diphtheria toxin receptor

Since M-MDCs can deliver H19 lncRNAs through exosomes, the next question we asked was whether the tumorigenicity of M-MDCs is also passed onto recipient cells along with H19 lncRNAs. Normal AT2 cells from 4-week-old ST, MPG-ST, or MPG-ST-iDTR mice were isolated and co-cultured with M-MDCs or their exosomes. Co-cultured normal AT2 cells did not acquire a malignant phenotype in subcutaneous cell transplantation (Fig. 5A). In addition, we isolated AT2 cells mingled with M-MDCs in lung metastases of MPG-ST-iDTR mice not subjected to DT treatment and examined their H19 expression and tumorigenicity. The results showed that these AT2 cells had a high expression of H19 (Fig. 5B) but did not grow into tumors in ectopic sites (Fig. 4A). These results suggest that the transfer of H19 through exosomes is a necessary but not sufficient condition for transforming AT2 cells.

We then explored the potential for H19 lncRNAs to endow AT-2 cells with a premalignant status. We further investigated the passage of several types of cells affected by H19 lncRNAs. The growth ability of primary AT2 cells from MPG-ST-iDTR mice younger than 16 weeks was limited. After the 6^th^ passage, the cells became larger, irregular in shape, gradually died, and thus could no longer be passaged. However, primary AT2 cells from lung metastases of MPG-ST-iDTR mice over 16 weeks old (without DT treatment) showed unlimited division and growth potential (Fig. 5C). Primary AT2 cells from MPG-ST-iDTR mice younger than 16 weeks ceased growing following 10 cumulative population doublings (CPDs). In contrast, the CPDs of AT2 cells from lung metastases of MPG-ST-iDTR mice over 16 weeks of age were 3-times higher, suggesting immortalization (Fig. 5D). In addition, the division and growth ability of normal AT2 cells co-cultured with the exosomes of M-MDCs was measured. Through immunoblotting analysis, we found a significant enhancement in the expression of telomerase reverse transcriptase (TERT) in AT2 cells from lung metastases of MPG-ST-iDTR mice (immortal AT2 cells) and normal AT2 cells co-cultured with the exosomes of M-MDCs (Fig. 5E). Telomerase activity assay reflected that immortal AT2 (I-AT2) cells and normal AT2 cells co-cultured with the exosomes of M-MDCs showed strong telomerase activity, whereas normal AT2 cells without H19 did not (Fig. 5F). Together, these data suggest that the introduction of H19 lncRNAs extended the lifespan of recipient cells and led to their immortalization.

### Alternative splicing of H19 lncRNA mediates the malignant transformation of AT2 cells

We can infer from the above results that H19 lncRNAs can only render a premalignant status of immortalization to AT2 cells, which allows us to continue to explore how the lung metastasis mingled AT2 cells acquire their tumorigenicity? Although H19 lncRNA is the reservoir of miR-675 [19], our results showed that miR-675 was not associated with the variation of Myc protein in M-AT2 cells (Additional file 9: Fig. S7D), demonstrating that the tumorigenicity of AT2 cells was not provided by H19 lncRNA regulated miR-675. Thus, we turn to try the alternative splicing of long noncoding RNAs [20, 21], which may suggest that the splice variant of H19 (Additional file 8: Fig. S8A) could confer AT2 malignancy.

**Fig. 6:**
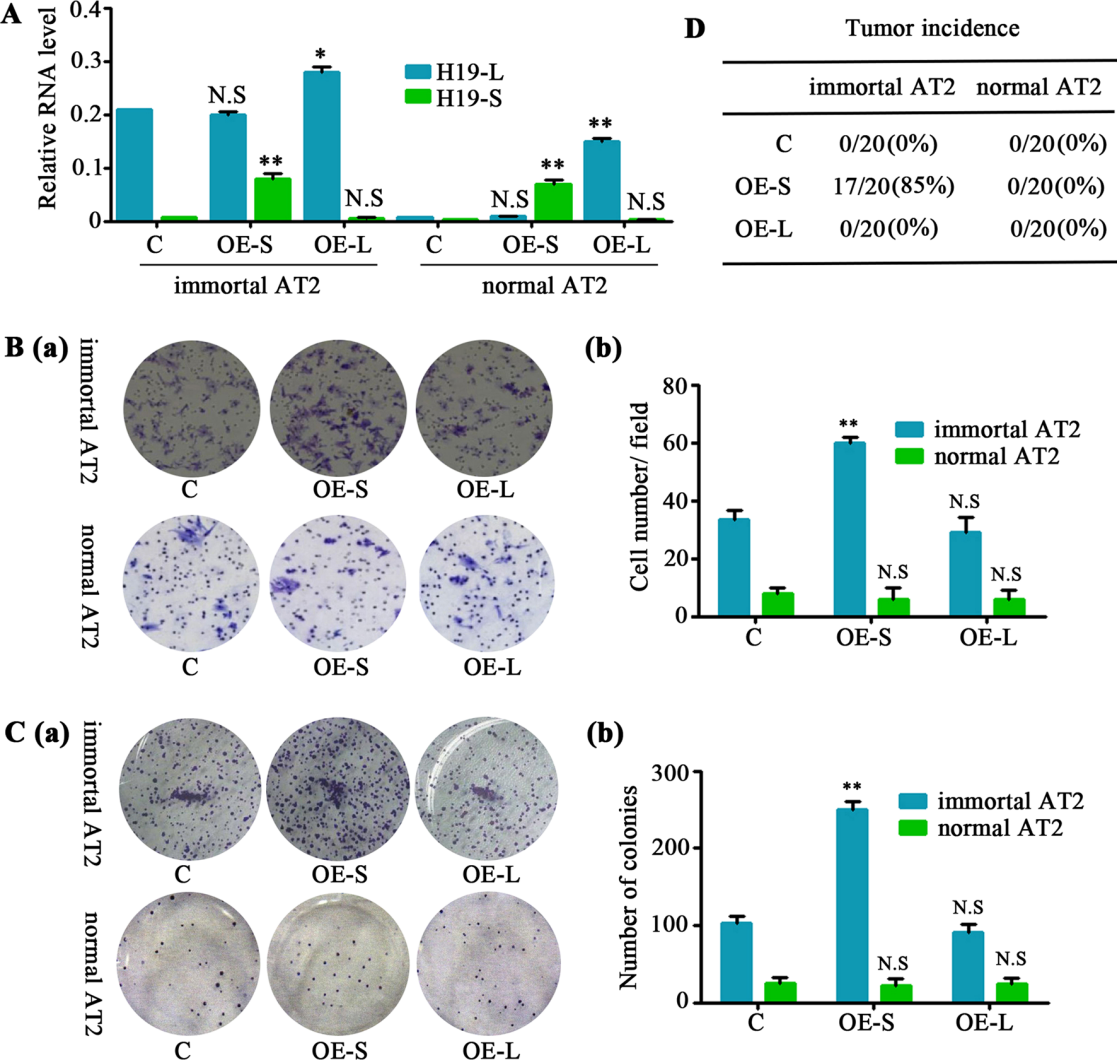
H19-S promotes tumorigenicity in immortalized AT2 cells. The tdTomato-positive AT2 cells were sorted from the peritumoral tissues (normal AT2 cells, *n* = 3) or tumor tissues (immortalized AT2 cells, *n* = 15) of the lung metastasis from 16-week-old MPG-ST-iDTR mice without DT injection. *n* = number of tissues. The plasmids including H19-L (full-length transcript) or H19-S (short transcript) were separately transfected into immortalized AT2 or normal AT2 cells. **A** qRT-PCR assessment of H19-L and H19-S in H19-L– or H19-S–overexpressing immortalized AT2 cells, normal AT2 cells, and their control cells. Data presented are the mean of triplicate experiments. Error bars indicate standard deviation (**P < 0.01; *P < 0.05; N.S, No significance). **B** Invasive capacities of the indicated cells were analyzed and quantified. Data presented are the mean of triplicate experiments. Error bars indicate standard deviation (**P < 0.01; N.S, No significance). **C** Colony-forming assays of H19-L or H19-S stably overexpressed in immortal AT2 cells, normal AT2 cells, and their control cells. Data presented are the mean of triplicate experiments. Error bars indicate standard deviation (**P < 0.01; N.S, No significance). **D** H19-L or H19-S stably overexpressed immortal AT2 cells, normal AT2 cells, and their control cells were subcutaneously transplanted into wild-type (WT) recipient mice. The tumor incidence, described as transplantation sites with tumors/transplanted mice amount (ratio), were measured at 18 weeks after transplantation. Abbreviations: C, plasmid control; OE-S, overexpression of H19-S; OE-L, overexpression of H19-L

Two PCR products of 429 and 298 bp were detected in M-AT2 cells. The bands were separately recovered and purified for subsequent sequencing analysis. We found that the 429-bp form corresponded to the full-length H19 transcript (H19-L) whereas the short form corresponded to a H19 transcript lacking exon 2 (H19-S) (Additional file 12: Fig. S8B). qRT-PCR further confirmed that H19-S was only highly expressed in M-AT2 cells (Additional file 12: Fig. S8C). Hence, we speculated that H19-S is critical for the malignant transformation of M-AT2 cells. To verify this, we introduced H19-S or H19-L into I-AT2 cells and normal AT2 cells. Overexpression of H19-S, rather than H19-L, in I-AT2 cells promoted their invasion and colony formation ability in vitro and tumorigenicity in vivo. However, transformation was not observed with the exogenous expression of H19-S or H19-L in normal AT2 cells (Fig. 6), indicating that H19-S can promote tumorigenicity only in immortalized AT2 cells.

**Fig. 7:**
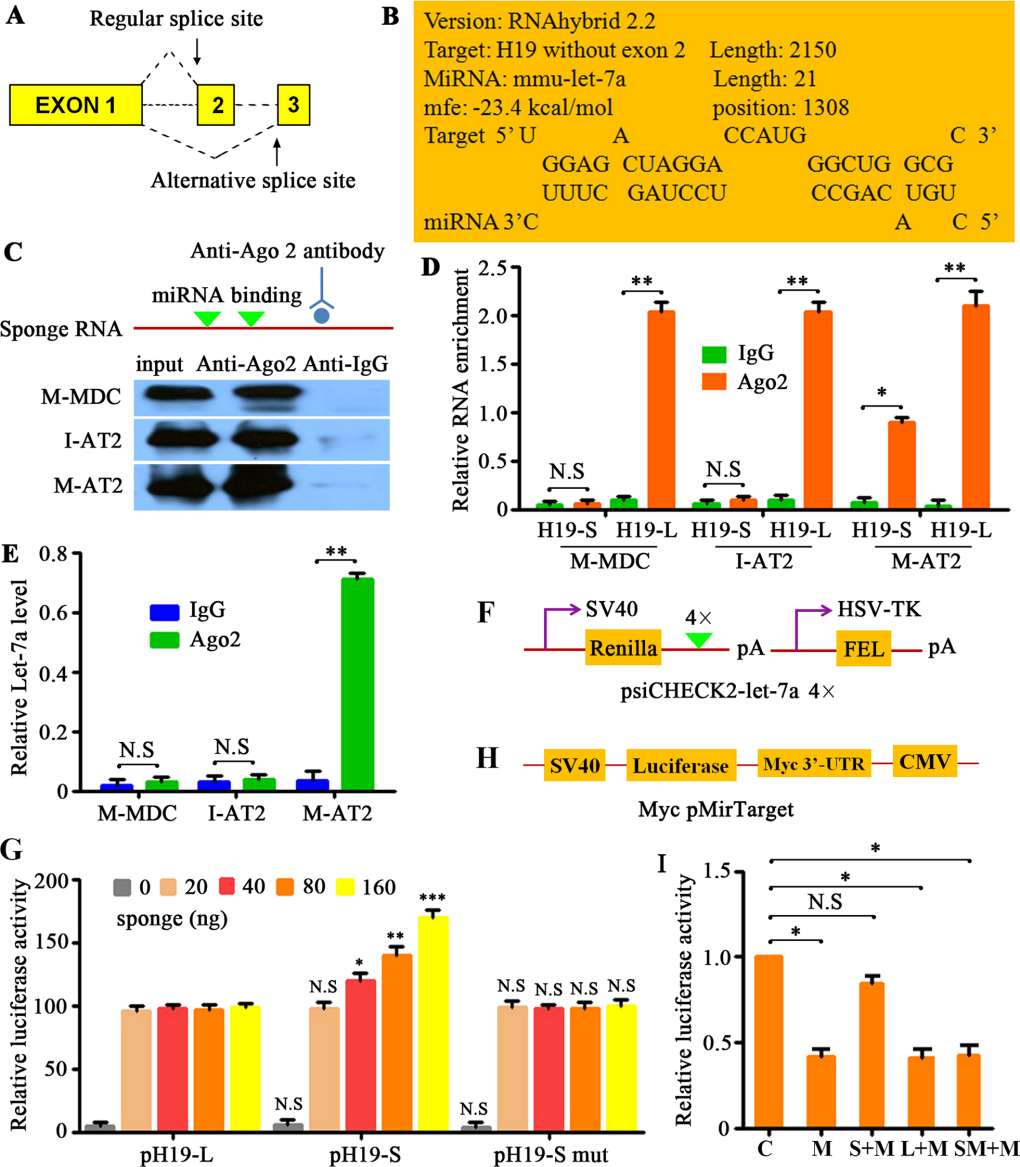
H19-S and Let-7a present in the same Ago2 complex in M-AT2 cells. **A** Schema for the alternative splicing of H19. **B** The online RNAhybrid tool predicted that the alternative splicing of H19 resulted in an emerging binding site for Let-7a. **C** Immunoprecipitation of Ago2 protein from cell lysates from M-MDC, I-AT2, and M-AT2 cells, with non-immune IgG control. The input lane indicates lysate samples used as a positive control for Ago2. **D** The tumor tissues (*n* = 13) of the lung metastasis were sorted for tdTomato-positive AT2 cells (I-AT2) or for GFP-positive MDC cells (M-MDC) from 16-week-old MPG-ST-iDTR mice without DT injection. The tdTomato-positive AT2 cells (M-AT2) were sorted from the lung metastasis (*n* = 5) of MPG-ST-iDTR mice two weeks after DT injection. qRT-PCR detection of H19-L or H19-S in M-MDC, I-AT2, and M-AT2 cells that were precipitated by Ago2 or negative control IgG and normalized to U6 snRNA. *n* = number of tumor tissues. Data presented are the mean of triplicate experiments. Error bars indicate standard deviation (**P < 0.01; *P < 0.05; N.S, No significance). **E** qRT-PCR detection of Let-7a endogenously associated with sponge RNA in M-MDC, immortal AT2 and M-AT2 cells that were pulled down by Ago2 or negative control IgG and normalized to U6 snRNA. Data presented are the mean of triplicate experiments. Error bars indicate standard deviation (**P < 0.01; N.S, No significance). **F** Scheme of Let-7a target luciferase reporter. **G** The luciferase activity assessment in normal AT2 cells transfected with sensor (psiCHECK2-let7a 4×), together with 0, 20, 40, 80, or 160 ng of sponge plasmid pH19-L, pH19-S or pH19-S mut. Data presented are the mean of triplicate experiments. Error bars indicate standard deviation (***P < 0.001; **P < 0.01; *P < 0.05; N.S, No significance). **H** Scheme of Myc pMirTarget luciferase reporter. **I** Activity of Myc pMirTarget luciferase reporter plasmids in N-AT2 cells co-transfected with Let-7a mimics or Let-7a mimics together with pH19-L, pH19-S, or pH19-S mut (normalized to control). C, control; M, mimics; S + M, pH19-S + mimics; L + M, pH19-L + mimics; SM + M, pH19-S mutant + mimics. Abbreviations: MDCs, mammary gland-derived cells; M-MDCs, malignant MDCs; M-AT2, malignant AT2 cells; I-AT2, immortalized AT2 cells

How does the H19-S transcript variant lead to the tumorigenicity of I-AT2 cells? We noticed that Myc expression was induced in M-AT2 cells (Fig. 4C), and this prompted us to investigate the Myc microRNA—Let-7a axis [17, 22]. The short alternative splice form of H19 (Fig. 7A) harbors a predicted binding site for Let-7a, beginning at nucleotide position 1,308 and ending at position 1,467 (Fig. 7B). We hypothesized that H19-S acts as a molecular sponge to sequester Let-7a, thereby inhibiting its targeting function on Myc [23]. RNA immunoprecipitation (RIP) assays using antibodies against mouse Ago2—a core component of the RNA-induced silencing complex (RISC) [24]—supported this sponging hypothesis. Ago2 antibody precipitated Ago2 protein-RNA complexes from M-MDC, I-AT2, and M-AT2 cell lysates (Fig. 7C), and endogenous H19-L or H19-S was preferentially enriched in Ago2 RIPs compared with control immunoglobulin G (IgG) antibody RIPs (Fig. 7D). Additionally, Ago2 RIP samples from M-AT2 cells were significantly enriched for endogenous Let-7a compared with those from M-MDCs or I-AT2 cells (Fig. 7E). From these findings, we infer that H19-S and Let-7a are present in the same Ago2 complex in M-AT2 cells.

To further explore whether H19-S may indeed act as a “sponge” to sequester Let-7a, normal AT2 cells, which exhibit a low amount of endogenous H19-L or H19-S (Fig. 6A), were transfected with the plasmids psiCHECK2-Let-7a 4× (encoding Let-7a binding sites; i.e., the “sensor” in this experiment) and various amounts of pH19-L or pH19-S (which expresses full-length mouse H19 or H19 without exon 2; i.e., the “sponge” in this experiment) (Fig. 7F). The relative luciferase activity increased in response to pH19-S in a dose-dependent manner (Fig. 7G), suggesting that ectopically expressed H19-S specifically sequestered endogenous Let-7a, thereby preventing it from inhibiting luciferase expression. Expression of H19-L or a mutant H19-S (pH19-S mut), in which the predicted Let-7a interaction sites were mutated, did not prevent Let-7a from binding to Rluc mRNA (Fig. 7G). These findings confirmed that Let-7a binding sites are required for this effect.

A luciferase reporter of the 3′ untranslated region (3’UTR) of Myc (Myc pMirTarget) was co-transfected into N-AT2 cells with Let-7a mimics or their controls. N-AT2 cells co-transfected with Let-7a mimics showed a significant decrease in the activity of luciferase constructs carrying the sequence of the Myc 3′UTR (Fig. 7H, I). The effects of H19-S or H19-L on the Myc luciferase reporter were also analyzed with pH19-L, pH19-S, and its mutant derivative, pH19-S mut. The relative activity of luciferase was rescued in pH19-S co-transfected cells but not in pH19-L or pH19-S mut co-transfected cells (Fig. 7H, I). Together, these data suggest that Myc is a target of Let-7a and that H19-S physically associates with Let-7a to function as a competing endogenous RNA (ceRNA) for Let-7a. H19-S modulates Let-7a availability by acting as a molecular sponge and thus inducing Myc expression.

**Fig. 8:**
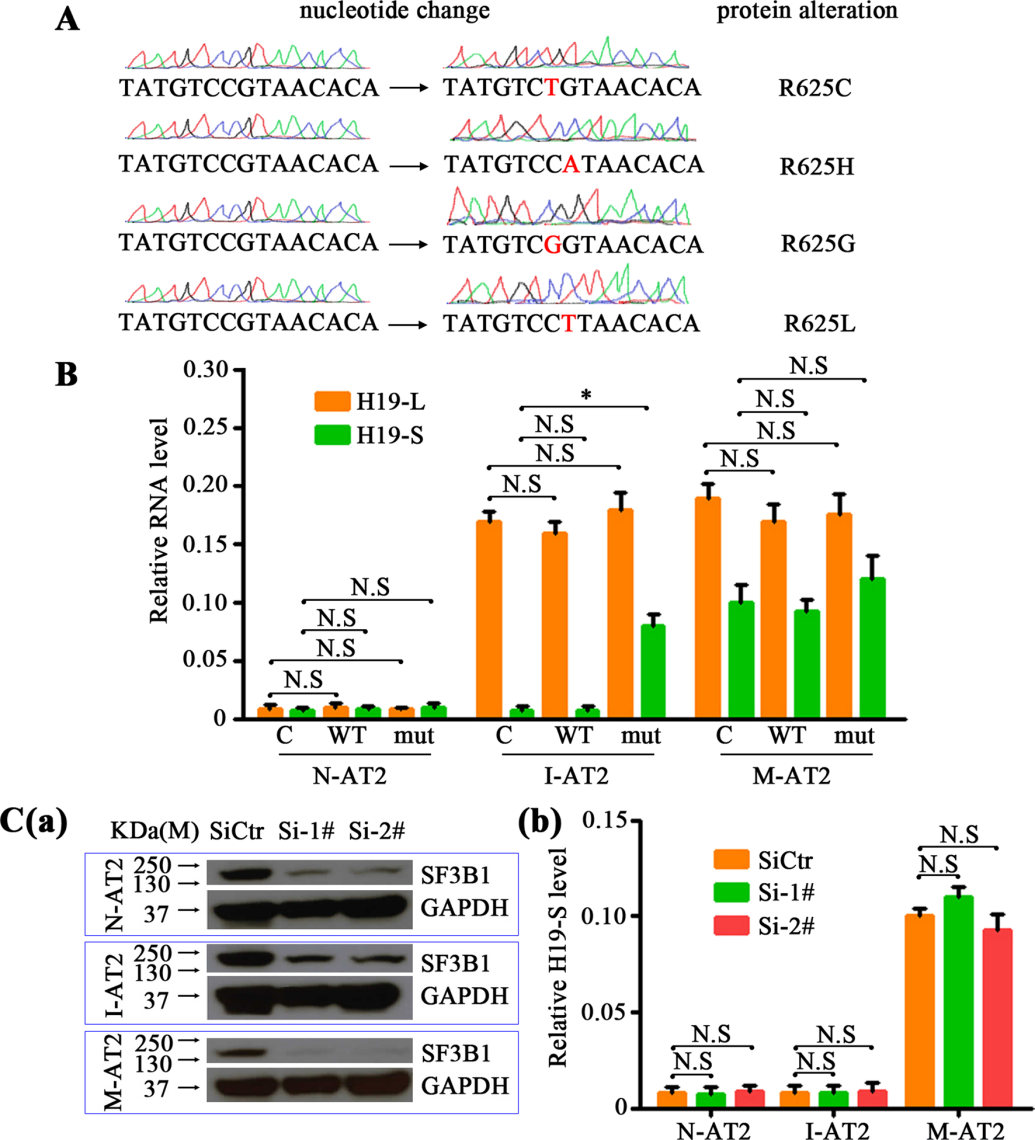
Mutant SF3B1-R625C, H19-S, and Let-7a mediate the malignant transformation of I-AT2 cells. **A** The lung metastases (*n* = 20) of MPG-ST-iDTR mice 2 weeks after DT injection were collected and sent for Sanger sequence traces of SF3B1-wild-type and SF3B1-mutant tumors. Representative mutations were showed. *n* = number of lung metastases. **B** N-AT2, I-AT2, and M-AT2 cells were transfected with the expression vectors of SF3B1-WT, SF3B1-mut (R625G) or their controls. The expression of H19-S or H19-L was analyzed by qRT-PCR. **C**. N-AT2, I-AT2 and M-AT2 cells were transfected with SF3B1 siRNAs or their control siRNAs. The expression of SF3B1 (**a**) or H19-S (**b**) was individually detected. Abbreviations: C, Control; WT, SF3B1-WT; mut, SF3B1-mut; SiCtr, SiRNA control; Si-4#, SF3B1 SiRNA-4#; Si-5#, SF3B1 SiRNA-5#; N.S, no significance; N-AT2, normal AT2 cells; I-AT2, immortalized AT2 cells; M-AT2, malignant AT2 cells; pH19-L, full-length mouse H19; pH19-S H19 without exon 2, i.e., the “sponge” in this experiment. *P < 0.05

Next, we investigated why H19 was alternatively spliced in malignant AT2 cells. The two most frequently mutated splicing factors are SF3B1, a core component of U2 snRNP, and SRSF2, a serine/arginine-rich (SR) protein that acts both in alternative and in constitutive splicing and interacts with U1 snRNP [25]. We amplified and sequenced SF3B1 and SRSF2 genes in normal AT2 and M-AT2 cells, and found that, while the SRSF2 gene was not mutated in any of these cells, SF3B1 had mutation at codon 625 in M-AT2 cells. We further manually examined the entire SF3B1 coding sequence of 114 lung metastases from twenty MPG-ST-iDTR mice with DT injection. Strikingly, all metastases had mutations and merely occurred at arginine-625, including fifty-nine R625H, thirty-five R625C, twelve R625G and eight R625L substitutions (Fig. 8A). The mode of action of this SF3B1 mutant was then addressed by constructing the expression vectors for the SF3B1-WT and one SF3B1 mutant R625C (SF3B1-mut). The different cell lines were transiently transfected with expression vectors for SF3B1-WT and SF3B1-mut and examined for the splicing of the endogenous H19. Overexpression of SF3B1-mut significantly increased the expression of H19-S in I-AT2 cells, whereas overexpression of SF3B1-WT had no effect on the alternative splicing of H19 (Fig. 8B).

To determine whether the SF3B1 mutation is a loss-of-function mutation, we assessed the effect of SF3B1 short-interfering RNA (siRNA)-mediated knockdown on alternative splicing in I-AT2, M-AT2, and N-AT2 cells. Non-targeting siRNA was used as a negative control, and siRNA-mediated knockdown was confirmed by immunoblotting. As shown in Fig. 8C, SF3B1 siRNA-mediated knockdown did not have any significant effect on the alternative splicing of H19 despite a 93% reduction in SF3B1 protein levels. These findings demonstrate that the SF3B1-mut splice pattern is not mimicked by SF3B1 knockdown. Together, our results provide evidence that the alternative splicing of H19 as H19-S in malignant AT2 cells was caused by SF3B1-R625C mutation.

Finally, AT2 cells infiltrating into lung metastasis were detected in metastatic breast cancer (MBC) patients. The immunohistochemical examination showed that cells expressing Sftpc protein scattered sporadically in metastatic lesions (Additional file 13: Fig. S9A). Meanwhile, compared with the adjacent tissues, Myc and H19 are highly expressed in metastatic cancer tissues, but the expression of H19-S has no significant change (Additional file 13: Fig. S9B), indicating that the AT2 cells infiltrating metastatic cancer in clinical samples are rare.

## Discussion

Using the classical MMTV-driven PyMT breast cancer model, we report in this study that breast epithelial cells with an ADH phenotype (E-MDCs) can be observed in the lungs at 4 weeks of age. By about 12 weeks, micrometastases appear in the lungs, exhibiting a malignant phenotype (M-MDCs). After another 3–4 weeks, macrometastases are detectable in the lungs. Surprisingly, lung metastases at this time contain lung alveolar AT2 cells. These infiltrating AT2 cells are immortalized by malignant M-MDC cells and can fully replace M-MDCs following their depletion by DT in DTR-expressing M-MDCs. A schematic diagram of these findings is presented in Additional file 14: Fig. S10.

Several highly metastatic cancer cells, including breast carcinoma cell lines (4T1 and MDA-MB-231), hepatocellular carcinoma cell lines (HCCLM3 and MHCC97-H), a nasopharyngeal carcinoma cell line (5-8F), and a lung cancer cell line (PGBE1), were selected and transfected with iDTR vectors (data not shown). When these iDTR-labeled cells were used to establish transplanted tumor metastatic models, we found that they could form distant organ metastasis, but these metastasis could not continue to grow in DT-treated mice. Further experiments also confirmed that 4T1-iDTR or other iDTR-labeled cells did not have a sufficiently high expression of H19 and could not immortalize normal AT2 cells (data not shown). These experiments strongly suggest that transplanted highly malignant cancer cells cannot reproduce an in situ generated cancer model. The essential difference may lie in the fact that MDCs must go through a premalignant (E-MDC) status before acquiring a malignant (M-MDC) status. It is intriguing to consider that the transformation of E-MDCs to M-MDCs not only promotes the abnormal expression of H19, Myc and perhaps other molecules but also initiates the immortalization of lung AT2 cells. The MMTV–PyMT cancer model recapitulates all the steps of breast cancer initiation and progression more so than established malignant cell lines. Our model, based on MMTV–PyMT mice, offers a unique opportunity to investigate the potential of early disseminated premalignant cells to initiate secondary tumors deriving from the colonized tissue, as suggested in a previous study [26], with exosomes inducing an oncogenic field effect.

Our results show that H19 expression increased in M-MDC cells (Fig. 4E). The detailed mechanism about how H19 being induced is not deeply investigated here. Our main concern is why AT2 cells infiltrating into metastatic cancer can evolve into malignant AT2 cells. Specifically, which pathway is involved into the upregulation of the proto-oncogene Myc in M-AT2 cells? It is noteworthy that even though M-MDCs can immortalize AT2 cells in the metastatic foci through H19 delivered by exosomes, only H19 lacking exon 2 (H19-S) can further transform immortalized AT2 cells (Fig. 6). At present, it is widely believed that cell immortalization may be a prelude to cell carcinogenesis [27]. However, immortalized cells have no tumorigenicity, and tumor cells have tumorigenic properties. They have many differences in molecular aspects such as activation of oncogenes, inactivation of tumor suppressor genes, and epigenetic mutations. Therefore, the immortal AT2 cells infiltrating into lung metastatic tumors can be seen as a special stage before their malignant transformation. Alternative splicing of H19 resulted in a new H19 splicer H19-S and a new let-7a sponge site (Fig. 7B). In other words, only H19-S rather than H19-L can competitively sponge let-7a, thereby eliminating the inhibition of let-7a on Myc expression. In our model, following DT treatment to eliminate M-MDCs, transformed AT2 cells harboring SF3B1 mutant generate H19-S as a competing endogenous RNA, selectively sponging let-7a, and allowing the expression of the proto-oncogene Myc (Fig. 8).

Breast cancer is a heterogeneous disease with multiple intrinsic tumor subtypes. These subtypes vary in tumor gene expression and phenotype and are most commonly grouped into four major subtypes: luminal A, luminal B, HER2-overexpressing and triple negative (or basal-like) [28]. According to different pathological types, breast cancer can be divided into: ductal carcinoma, lobular carcinoma, eczematoid carcinoma, medullary carcinoma, papillary carcinoma, mucinous adenocarcinoma, squamous cell carcinoma, etc. [29]. The pathological complexity of MMTV–PyMT mice is far less than that of real human cancer tissue. The spontaneous tumors of MMTV–PyMT female mice are all invasive ductal carcinoma [30, 31]. Thus, our model based on MMTV–PyMT mice probably reflects merely one subtype of breast cancer in clinical patients.

Although our model does not represent all clinical metastatic breast cancer, our experimental results show that the regenerative cancer after the removal of lung metastatic cancer is formed by lung AT2 cells rather than breast derived cells. This may partly explain the onset of resistance to the standard of care in clinical practice. Mortality associated with metastasis is mainly due to the failure for standard therapies because of gene mutations [32]. Our model proved that SF3B1 bearing mutations at arginine-625 was the initiator of regenerative growth of metastasis. One way to observe the regenerative growth of metastasis in clinical samples is to examine patients with recurrent tumors after surgical resection of metastases. Unfortunately, except for a few cancer patients such as colorectal cancer with liver metastasis, patients with metastasis rarely undergo surgery, and there are few cases of recurrence after metastatic cancer surgery. All these practical difficulties seriously hinder the possibility of clinical verification of our experimental results.

In conclusion, we identified an unexpected oncogenic process that can contribute to the regenerative growth of metastatic carcinoma. Although this mechanism may be hard to document in human metastatic tumors at this stage, our results suggest that clinically, the origin of metastasis does not necessarily derive from the primary site, and the treatment of metastatic cancer should not only target cells derived from the primary site. It should call for more attention about such specific origin of metastatic tumor.

## Materials and methods

### Mice

Mice lines *MMTV-Cre* (jax stock#003553), *R26-(CAG-LSL-zsGreen)*, *R26-LSL-DTR, MMTV–PyMT* (jax stock#022974), *H11-(CAG-RSR-tdTomato)* were provided by Shanghai Model Organisms. The new knock-in mouse line *Sftpc-e(IRES-Dre-pA)1* was generated by homologous recombination using CRISPR/Cas9 technology.

### Whole-mount fluorescence microscopy

Tissues were washed in cold phosphate-buffered saline (PBS), and fixed in 4% paraformaldehyde (PFA) at 4 °C for 1 h, followed by three rounds of washing with PBS (15 min each time). Tissues were placed on an agar gel for whole-mount bright-field or fluorescence imaging using a Zeiss stereo microscope (AxioZoom V16).

### H&E staining

Briefly, 10-μm-thick cryosections collected from different organs or tissues were incubated in Hematoxylin A solution for 3 min. Sections were washed 3 times with water, rinsed in 1% concentrated HCl diluted in 70% ethanol for 1 min, and then again washed 3 times with water. Sections were then incubated in 1% ammonia water for 1 min, washed 3 times, and then stained with Eosin-Y solution for 8–10 s before dehydration in a series of ethanol and xylene. Finally, sections were mounted with neutral balsam. All imaging data were acquired using an Olympus microscope (Olympus, DP72).

### Immunostaining and immunohistochemistry

Immunohistochemistry (IHC) was performed as previously described [16]. Briefly, tissues were collected in cold PBS and then fixed in 4% PFA at 4 °C for 1 h. After 3 PBS washes, tissues were placed in a 30% sucrose/PBS solution overnight at 4 °C, then embedded in the OCT compound (Sakura) and stored at − 80 °C until sectioning. Cryosections, 10-μm-thick, were collected on positively charged slides and stored at − 20 °C until use. For immunostaining, tissue sections were blocked with PBSST (0.1% Triton X-100/2.5% normal donkey serum/PBS) for 30 min at room temperature and then incubated with primary antibody overnight at 4 °C. Tissue sections were then washed with PBS three times and then incubated with AlexaFluor-conjugated secondary antibodies (Invitrogen) for 30 min at room temperature. Sections were then washed with PBS three times and mounted with mounting medium containing DAPI. For weak signals, horseradish peroxidase or biotin-conjugated secondary antibodies and a tyramide signal amplification kit (PerkinElmer) were used. The antibodies used were as follows: tdTomato (Rockland, 600-401-379; 1:1000 dilution), GFP (Nacalai Tesque Cat# 04404-84, RRID:AB_10013361; 1:500 dilution), PyMT (Abcam, 15,085; 1:500 dilution). Images were acquired using a Nikon confocal microscope (Nikon A1) or a Zeiss confocal microscope (LSM 710). Images were analyzed using Image J and Photoline software. Quantitation was performed by dividing the number of green or red fluorescent cells in the tumor area by the number of blue (DAPI-stained) cells.

### Animal studies

All mice were housed under standard conditions. The indicated cells were trypsinized, washed with PBS, and suspended in DMEM without serum. A total of 2 × 10^6^ cells were subcutaneously injected into the right back flanks or orthotopically injected into the right mammary glands of syngeneic WT mice. Tumor growth was measured every 4 or 5 days, and tumor volume was estimated as 0.52 × length × width. Tumors were harvested from ether-anesthetized mice. Histopathological confirmation for tissue sections were conducted by pathologist Dr. Jing-Yu Wang (Affiliated Hospital of Jiaxing University). All procedures were approved by a Medical Ethics committee of Jiaxing University.

### RNA extraction and quantitative real-time PCR

Total RNA from indicated cells was isolated using TRIZOL (Invitrogen) and 1 μg total RNA was used for reverse transcription. Quantitative Real-time PCR (qRT-PCR) was subsequently performed in triplicate with a 1:4 dilution of cDNA using the SYBRH Green RT-PCR Reagents Kit (Roche). Primers for H19-L, H19-S, and Myc are listed in Additional file 15: Data file S3. The comparative Ct method was used to compute relative expression values. The results were normalized to U6 snRNA, as indicated.

### RNA sequencing and data analysis

Exosomes from M-MDC and E-MDC cells were used for RNA-seq. Total RNA was extracted using TRIzol reagent (Invitrogen) according to the manufacturer’s instructions. The RNA was qualified by Agilent 2100 bioanalyzer (Thermo Fisher Scientific, Waltham, MA, USA), after which the qualified RNA was subjected to remove rRNA through a Ribo-Zero Magnetic Kit (Epicentre). RNA libraries were constructed by the Epi™ mini long RNA-seq kit, and the Illumina NovaSeq 6000 was used as the instrument model. The DESeq2 algorithm was applied to filter the differentially expressed genes, following the significant analysis and false discovery rate (FDR) analysis.

### Single-cell RNA sequencing

The total 30 lung metastatic tumors from 16 weeks MPG mice were separately collected and digested as single cells for FACS sorting by the green laser (561 nm, 100 mV). The positive fluorescent cells were discarded, and non-fluorescent cell suspension were FACS sorted out CD3 positive cells. The remaining cells after two round FACS sorting (infil) or single cells from one peritumoral lung tissue of 16 weeks MPG mice (peri) were, respectively, loaded onto the chromium single-cell controller (10× Genomics) to generate single-cell gel beads in the emulsion according to the manufacturer’s protocol. Reverse transcription was performed on a S1000TM Touch Thermal Cycler (Bio Rad) at 53 °C for 45 min, followed by 85 °C for 5 min, and hold at 4 °C. The cDNA was generated and then amplified, and quality assessed using an Agilent 4200 Single-cell RNA-Seq library preparation. According to the manufacture’s introduction, single-cell RNA-seq libraries were constructed using Single Cell 5’ Library and Gel Bead Kit. The libraries were sequenced using an Illumina Novaseq6000 sequencer with a sequencing depth of at least 77,618 reads per cell with pair-end 150 bp (PE150) reading strategy.

### MTT assay

The indicated cells were plated at 5000 cells/well in multiple 96-well plates, and MTT solution (Thiazolyl Blue Tetrazolium Bromide, Sigma, St. Louis, USA) then was added to separate plates after 24, 48, 72 or 96 hour of growth. Dimethyl sulfoxide (DMSO, Sigma, St. Louis, USA) was added at 100 μl/well and plates were incubated for 20 min in a 37 °C incubator. Absorbance at a wavelength of 570 nm was measured with a microplate reader (Bio-Rad, CA, USA).

### Patient eligibility

This study included 28 metastatic breast cancer (MBC) patients treated at Lishui City People’s Hospital from August 2020 to December 2021. Patients meeting all the following requirements were eligible for enrollment: (i) a diagnosis of invasive breast cancer and lung metastasis confirmed by histology, (ii) no treatment before diagnosis, and (iii) provision of voluntary written informed consent. Human specimens included metastatic tumor tissue and adjacent normal tissue (beyond tumor margins) from MBC patients for analysis of the expression of the H19-L, H19-S and Myc.

### Ago2 RNA immunoprecipitation (RIP) assay

To determine whether the lncRNA H19 and let-7a are associated with the RNA-induced silencing complex (RISC), we performed an RNA pull-down assay using Ago2 antibody to precipitate the complex and detect H19 or miRNAs from the pellet using qRT-PCR. Briefly, M-MDC, I-AT2 and M-AT2 cells were rinsed with cold PBS and fixed with 1% formaldehyde for 10 min. After centrifugation, cell pellets were collected and resuspended in NP-40 lysis buffer supplemented with 1 mM phenylmethylsulfonyl fluoride (PMSF), 1 mM dithiothreitol, 1% protease inhibitor cocktail (Sigma-Aldrich), and RNase inhibitor (200 U/ml) (Life Technologies). The cell lysates were stored at − 80 °C before use. The supernatant from the cell lysates was collected by high-speed centrifugation. To generate antibody-coated beads, protein G Sepharose 4 Fast Flow bead slurry (GE Healthcare) was rinsed with NT2 buffer (50 mM Tris–HCl, 150 mM NaCl, 1 mM MgCl_2_, and 0.5% NP-40) and incubated with an antibody against Ago2 (Abcam). Non-immune mouse IgG (Sigma-Aldrich) was used as a negative control. For the RIP assay, the supernatant was incubated with antibody-coated Sepharose beads overnight. The beads were rinsed with cold NT2 buffer, and then incubated with proteinase K (10 mg/ml) (Sigma-Aldrich). The RNAs bound to the Ago2 antibody were purified with an RNeasy Mini Kit (Qiagen) and subjected to qRT-PCR.

### In vitro invasion and colony formation assays

The invasive ability of overexpressed H19-L or H19-S in I-AT2 or normal AT2 cells and their corresponding control cells was measured using 24-well Transwell plates (8-mm pore size; Corning), as previously described [16]. Briefly, cells (5 × 10^4^) were suspended in 200 ml DMEM and then added to the upper chamber of the Transwell with a noncoated membrane. The chamber inserts were coated with Matrigel (BD Biosciences) at 1:7 dilution. For colony forming assays, overexpressed H19-L or H19-S in I-AT2 or normal AT2 cells and their corresponding control cells (1 × 10^3^ cells each) were seeded into the wells of a 6-well plate. After culturing for 12 days, cells were fixed with 70% ethanol and then stained by 0.2% cresyl violet solution. The images were captured, and the colonies were counted.

### Plasmids and siRNAs

psiCHECK2-let-7 4 × (#20,930) plasmids were obtained from Addgene. SF3B1 mouse open reading frame clone (#Mm35243) was purchased from GeneCopoeia. H19 mouse open reading frame clone (#MC207056), SF3B1 mouse siRNA oligo duplex (#SR422625), Cloning vector PCMV6-Kan/Neo (#PCMV6KN) and pMirTarget 3′-UTR luciferase assay vector (#PS100062) were purchased from OriGene Technologies. The mouse let-7a miRNA mimics (stem-loop accession number: MI0000556) were purchased from Ambion/Life Technologies. To make Myc pMirTarget luciferase reporter, 3′-UTR fragments (Additional file 15: Data file S3) of Myc were digested by restriction enzyme SgfI and MluI and cloned into the pMirTarget cloning vector. To make psiCHECK2-let-7a 4 × , an annealed oligonucleotide fragment containing copies of let-7a binding sites (Additional file 15: Data file S3) was inserted into psiCHECK2-let-7 4× opened with XhoI and NotI. To make pH19-L, PCR was carried out using a H19 mouse open reading frame clone as a template. To make pH19-S and pH19-S mut, gene splicing by overlap extension PCR (SOE-PCR) was carried out using pH19-L as a template with primers (Additional file 15: Data file S3). The resulting PCR fragments were digested and ligated to pCMV6-Kan/Neo opened with HindIII and XhoI. To make SF3B1-WT constructs, PCR was carried out using an SF3B1 mouse open reading frame clone as a template. To make SF3B1 mut, Arginine 625 codon of SF3B1 in the SF3B1-WT plasmid was mutated to cysteine using the TaKaRa MutanBEST Kit with one mutation-introducing primer and one 5′ base adjacent corresponding primer (Additional file 15: Data file S3). The resulting PCR fragments were inserted into the BamHI and EcoRV sites of the pcDNA3.1 vector (RRID: Addgene 79663), respectively.

### Exosome collection

E-MDC, M-MDC, N-AT2, and M-AT2 cells were sorted by FACS and rinsed with PBS twice. Exosomes were isolated using sequential centrifugation at 4 °C: at 2000×*g* for 15 min to remove cell debris, and then at 100,000×*g* for 3 h to pellet the exosomes. Pellets were resuspended in PBS and then recentrifuged at 100,000×*g* for 3 h to obtain purified exosomes. The purified exosome pellet was resuspended in PBS and adjusted according to the final cell number to yield an equivalent secreted exosome concentration on a per cell basis.

### Mammary gland-derived cells clearance

For mammary gland-derived cells ablation in MPG-ST-iDTR mice, diphtheria toxin (DT; #D0564, Sigma-Aldrich) administration was performed, as described previously, with some modifications [13]. In brief, adult mice were administered with intraperitoneal injections of DT (100 ng/mouse) with one of the following regimens: once a day for 3 consecutive days, 3 times a day for 3 consecutive days, or 3 times a day for 7 consecutive days. Their littermates bearing Cre recombinase were also administered with DT and used as control. No specific adverse side effects of DT were observed when administered to the control and iDTR mice. At 12 h after the final DT injection, mice received a single intraperitoneal injection of LPS (2.5 mg/kg, #L2630, Sigma-Aldrich).

### Telomerase activity assay

Telomerase activity was determined by radioactive telomeric repeat amplification protocol (TRAP) assay, as described previously [33], using cell lysates equivalent to approximately 5000 cells or heat-inactivated samples and polymerase chain reaction (PCR) amplification for 27 cycles. The PCR products were separated in nondenaturing polyacrylamide gels by electrophoresis.

### PCR array

Lung metastatic M-AT2 cells and parenchyma N-AT2 cells from MPG-ST-iDTR mice after DT treatment were isolated by FACS, and RNA was extracted using an RNeasy Plus Mini Kit (QIAGEN). RNA was converted to cDNA using a cDNA Synthesis Kit (QIAGEN). We profiled 84 oncogenes and tumor suppressor genes using Oncogenes and Tumor Suppressor Genes PCR Array (YINGBIOTECH, #37,125-62,660-72,351).

### Western blotting

Cells were harvested in lysis buffer containing Complete Protease Inhibitor Cocktail (Roche) and subjected to SDS-PAGE. Cellular proteins were transferred onto a nitrocellulose membrane (Millipore) and probed for anti-TERT (#ITA7204, Geno Technology, Inc.; dilution 1:1000), anti-Myc (LSBio [LifeSpan] Cat# LS-B2791-50, RRID:AB_1934317; dilution 1:1000), anti-CD9 (Santa Cruz Biotechnology Cat# sc-13118, RRID:AB_627213; dilution 1:2000), anti-CD63 (Santa Cruz Biotechnology Cat# sc-5275, RRID:AB_627877; dilution 1:2000), anti-CD81 (Santa Cruz Biotechnology Cat# sc-166029, RRID:AB_2275892; dilution 1:2000) or anti-GAPDH (#9484, Abcam; dilution 1:5000) antibodies. After washing, the membranes were incubated with secondary antibodies. The reaction was revealed using an ECL chemiluminescence kit (Amersham Biosciences) with Eastman Kodak Co. hyperfilm and quantified by Multi Gauge software (Fujifilm Life Sciences). Data are presented as the mean ± SD, based on at least three independent repeats.

### DNA sequencing

PCR products were subjected to agarose gel electrophoresis. The target bands were cut, recovered, and purified. Two-dimensional sequencing was performed by Shanghai Huanuo Biotechnology Company on an ABI prism 377 DNA sequencer. The sequencing results were compared and analyzed in GenBank.

### Statistics

All mice were randomly assigned to different experimental groups. All data were determined from 5 independent experiments (as indicated in each figure legend) and are presented as mean values ± SEM. Differences between groups were analyzed using a Student’s *t* test. The differences were deemed statistically significant at *P* < 0.05.

## Supplementary Information

**Additional file 1**. **Figure S1**: Generation of mouse models. **A** The #1 MMTV-Cre mouse was mated with the #2 LSL Reporter mouse) to produce the #3 MMTV-Green mouse, whose mammary gland cells were labeled with green fluorescence. The LoxP-stop was removed by Cre recombinase. The #3 MMTV-Green mouse was then bred with #4 MMTV-PyMT mouse to obtain the #5 MMTV-PyMT-Greenmouse. Meanwhile, the #6 Sftpc-Dre mouse was crossed with the #7 RSR Reporter mouse) to obtain the #8 Sftpc-tdTomatomouse. After that, the #5 GFP-labeled breast cancer MPG mouse was mated with the #8 ST mouse to make the #9 MPG-ST mouse. Finally, the #11 MPG-ST-iDTR mouse was obtained by mating the #9 MPG-ST mouse with the #10 Cre-inducible diphtheria toxin receptortransgenic micemouse. **B**GFP-positive FACS-sorted cells from various sources mice were subcutaneously or orthotopically transplanted into syngeneic wild-type recipient mice; sources of cellswere from breast ADH tissue, or lungharvested from 4-week-old MPG mice; from lung micrometastasesof 12-week-old MPG mice; and from metastatic fociof 16-week-old MPG mice. Tumor incidence, described in orthotopic and subcutaneous transplantation sites with tumors/transplanted mice amount, was measured at 18 weeks after transplantation. **C.** Immunofluorescence detection for MDCs in lung. Br, bronchus. Scale bar, 200 μm.GFPand PyMTdouble immunofluorescence for MDCs in bone marrow. Scale bar, 20 μm.Scatter dot-plot displaying numbers of GFP+ and PyMT+ cells per 50,000 bone marrow cells.

**Additional file 2**. **Figure S2**: Exclusion on non-specific activation of MMTV in the lungs. The lung of 18-week-old MPGor MMTV-Greenmice were collected and detected by fluorescent microscope. Scale bar, 700 μm.

**Additional file 3**. **Figure S3**: Construction of Sftpc-Dre mouse. **A** Schematic diagram of design strategy. CRISPR/cas9 technology was used to knock in the IRES-Dre-pA expression boxat exon 6 site of Sftpc gene by homologous recombination. **B** Constructionand identificationof the donor vector. Cas9 mRNA and gRNA were obtained by in vitro transcription. The In-Fusion cloning method was used to construct the donor vector, which contained 2.9 kb 5’ homologous arm, IRES-Dre-pA and 3.0 kb 3’ homologous arm. **C** Identification strategy of F0 generation mice. The F0 generation mice were obtained by microinjection of Cas9 mRNA, gRNA and donor vector into the fertilized eggs of C57BL/6J mice. The 5’ homologous recombination-positive genome should amplify the 4.6-kb fragment, whereas the negative genome has no fragment; the 3.1-kb fragment should be amplified from the 3’ homologous recombination-positive genome, and a 6.9-kb fragment should be amplified from the negative genome. **D** PCR identification results of positive F0 generation mice. The F0 generation mice with positive double arm homologous recombination were No.18 and No. 23. Number, F0 mouse number; WT, wild-type control; M, 1 kb DNA marker. **E** The 5' and 3' homologous arms of F1 generation mice were identified by PCR. F0 generation mice are chimeric; they do not necessarily have the ability of stable inheritance. Therefore, these F0 generation mice need to be subcultured to obtain stable F1 generation mice. F0 generation positive mice were mated with wild-type C57BL/6J mice to obtain F1 generation mice. **F** The alignment between the target sequences and the PCR products of the F1 generation mice. Query, target sequence1 recombinant genomic DNA sequence); Subject, sequencing result of PCR products; Red underline, the 5’ or 3’ homologous arm sequence; Blue underline, knock in sequence.

**Additional file 4**. **Figure S4**: Label mice with two recombination systems. The mammary gland and lung of 3-week-old MPG-ST mice were collected and cut into slices for detection under fluorescence microscope. Representative images were showed. n = number of mice. Scale bar, 320 μm.

**Additional file 5**. **Table S1**: Lung metastasis after DT treatment

**Additional file 6**. **Figure S5**: The regenerative growth of metastasis after DT injection. MPG-ST-iDTR miceat 16 weeks were injected with 100 ng diphtheria toxinevery 8 h for 7 consecutive days. Lung metastases were detected by fluorescence microscope. Representative images of lung sections without DT treatmentand at 1 weekor 2 weeksafter DT injection. Circle mark indicate the tumor site. n = number of mice groups. Scale bar, 320 μm

**Additional file 7**. **Figure S6**: AT2 cells from the new generated tumor are malignant. MPG-ST-iDTR miceat 16 weeks were injected with 100 ng diphtheria toxinevery 8 h for 7 consecutive days. After DT injection for 2 weeks, AT2 cells among the lung metastasis were FACS-sorted and subcutaneously transplantedinto syngeneic wild-type mice. H&E and immunohistochemistry for adenocarcinoma markerswere applied after growth for 18 weeks. Magnification, 4×, 10×and 20×. n=number of mice.

**Additional file 8**. Data file S1: PCR array plate layout.

**Additional file 9**. **Figure S7**: PCR array and exosomes detection. **A** Transmission electron microscopy of exosomes secreted by malignant MDCs. Scale bar, 100 nm. **B** Classic exosome biomarkers were detected by western blotting. Quantification of relative protein level. **C** E-MDC, M-MDC, N-AT2and M-AT2cellswere collected. Their P53 protein expression were detected by western blotting. Data were quantified and presented as the mean ± SD of triplicate experiments. **D** The tdTomato-positive cellswere FACS sorted from the lung metastasis of the MPG-ST-iDTR mice 2 weeks after DT treatment and transfected with miR-675 mimics or antagomir. The RNA expression of Smad 1and Mycwas measured and quantifiedby qRT-PCR. Data presented are the mean of triplicate experiments. Error bars indicate standard deviation. N.S, no significance; *, p<0.05; **, p<0.01.

**Additional file 10**. Data file S2: List of all the expressed long ncRNAs identified by RNA-seq in the exosomes from E-MDC and M-MDC cells.

**Additional file 11**. **Table S2**: The highly expressed LncRNAs in the exosomes from M-MDC cells.

**Additional file 12**. **Figure S8**: Alternative splicing of H19 lncRNA in malignant AT2 cells. **A** Schemes illustrating PCR amplification of H19 variants. E1 and E2 indicate PCR primers for H19 cDNA. **B** RT-PCRand sequenceanalysis for H19 transcript using E1 and E2 primers. M, DNA ladder; Lane 1: E-MDCs; Lane 2: M-MDCs; Lane 3: N-AT2 cells; Lane 4: M-AT2 cells. **C** The RNA level of H19 without exon 2 in indicated AT2 cells were analyzed by qRT-PCR and normalized to U6 snRNA. Data presented are the mean of triplicate experiments. Error bars indicate standard deviation. H19-L, full length H19; H19-S, H19 without exon 2. Abbreviations: MDCs, mammary gland-derived cells; E-MDCs, early MDCs; M-MDCs, malignant MDCs; N-AT2 cells, normal AT2 cells; M-AT2 cells, malignant AT2 cells.

**Additional file 13**. **Figure S9**: Investigation in clinical samples. **A** H&Eor IHCfor Sftpc were applied onto the slices of lung metastasis from MBC patients. Arrows show the Sftpc positive cells. left panel: scar bar, 320um; magnification, 1×. middle panel: scar bar, 80um; magnification, 4×. right panel: scar bar, 40um; magnification, 10×. **B** The RNA expression of H19-L, H19-S or Myc in lung metastasesand their paired adjacent tissuesof 28 MBC patients were analyzed by qRT-PCR. The results were presented by M/A expression and normalized to U6 snRNA.

**Additional file 14**. **Figure S10**: Summary of the regenerative growth of metastasis. Schematic diagram of metastasis and regenerative growth of metastasis.

**Additional file 15**. Data file S3: Sequences and primers.

## Data Availability

The RNA-seq datasets generated for this study can be found in the Gene Expression Omnibus under accession GSE222524. The authors declare that all other data supporting the findings of this study are available within the paper and supplementary information files.

## Abbreviations

MMTV: Mouse mammary tumor virus
PyMT: Polyomavirus middle T antigen
LTR: Long terminal repeat
MPG: MMTV–PyMT-Green
ADH: Atypical ductal hyperplasia
WT: Wild-type
MDCs: Mammary gland-derived cells
E-MDC: Early MDCs
M-MDCs: Malignant MDCs
AT2 cells: Alveolar type II cells
Sftpc: Surfactant protein C
ST mice: Sftpc-tdTomato mice
MPG-ST mice: MMTV–PyMT-Green/Sftpc-Tomato mice
DTR: Diphtheria toxin receptor
DT: Diphtheria toxin
M-AT2: Malignant AT2
N-AT2: Non-malignant AT2
I-AT2: Immortal AT2
CPDs: Cumulative population doublings
TERT: Telomerase reverse transcriptase
H19-L: Full-length H19 transcript
H19-S: H19 transcript lacking exon 2
RISC: RNA-induced silencing complex
ceRNA: Competing endogenous RNA
SR: Serine/arginine-rich
