# Supplementary figures and images for: Breast metastatic tumors in lung can be substituted by lung-derived malignant cells transformed by alternative splicing H19 lncRNA
Source: Breast Cancer Res. 2023 May 30;25:59. doi: 10.1186/s13058-023-01662-z (PMC10228081; doi:10.1186/s13058-023-01662-z)

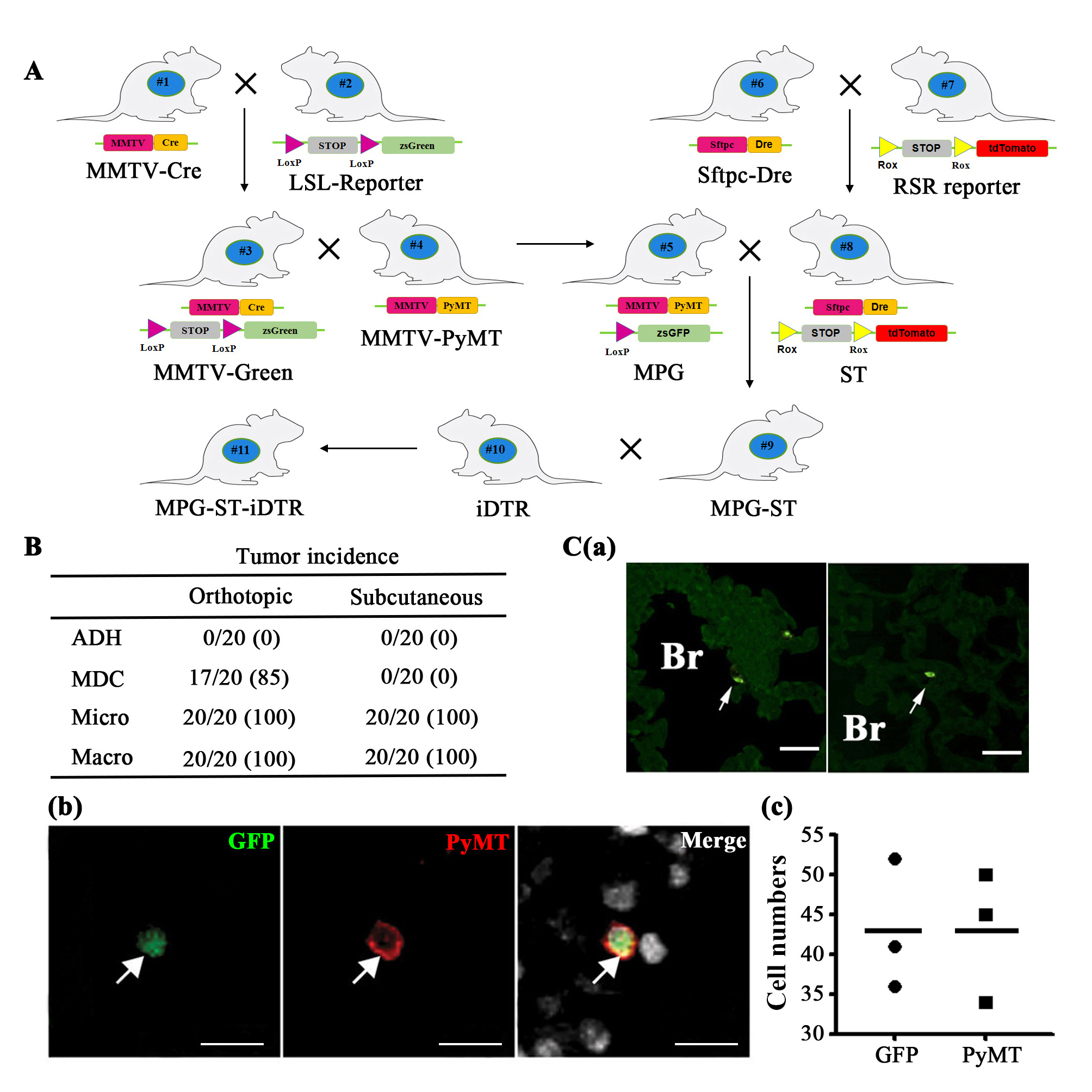

Supplement: Supplementary file 1 — Additional file 1. Figure S1: Generation of mouse models. A The #1 MMTV-Cre mouse was mated with the #2 LSL Reporter mouse) to produce the #3 MMTV-Green mouse, whose mammary gland cells were labeled with green fluorescence. The LoxP-stop was removed by Cre recombinase. The #3 MMTV-Green mouse was then bred with #4 MMTV-PyMT mouse to obtain the #5 MMTV-PyMT-Greenmouse. Meanwhile, the #6 Sftpc-Dre mouse was crossed with the #7 RSR Reporter mouse) to obtain the #8 Sftpc-tdTomatomouse. After that, the #5 GFP-labeled breast cancer MPG mouse was mated with the #8 ST mouse to make the #9 MPG-ST mouse. Finally, the #11 MPG-ST-iDTR mouse was obtained by mating the #9 MPG-ST mouse with the #10 Cre-inducible diphtheria toxin receptortransgenic micemouse. BGFP-positive FACS-sorted cells from various sources mice were subcutaneously or orthotopically transplanted into syngeneic wild-type recipient mice; sources of cellswere from breast ADH tissue, or lungharvested from 4-week-old MPG mice; from lung micrometastasesof 12-week-old MPG mice; and from metastatic fociof 16-week-old MPG mice. Tumor incidence, described in orthotopic and subcutaneous transplantation sites with tumors/transplanted mice amount, was measured at 18 weeks after transplantation. C. Immunofluorescence detection for MDCs in lung. Br, bronchus. Scale bar, 200 μm.GFPand PyMTdouble immunofluorescence for MDCs in bone marrow. Scale bar, 20 μm.Scatter dot-plot displaying numbers of GFP+ and PyMT+ cells per 50,000 bone marrow cells. [file 13058_2023_1662_MOESM1_ESM.jpg]

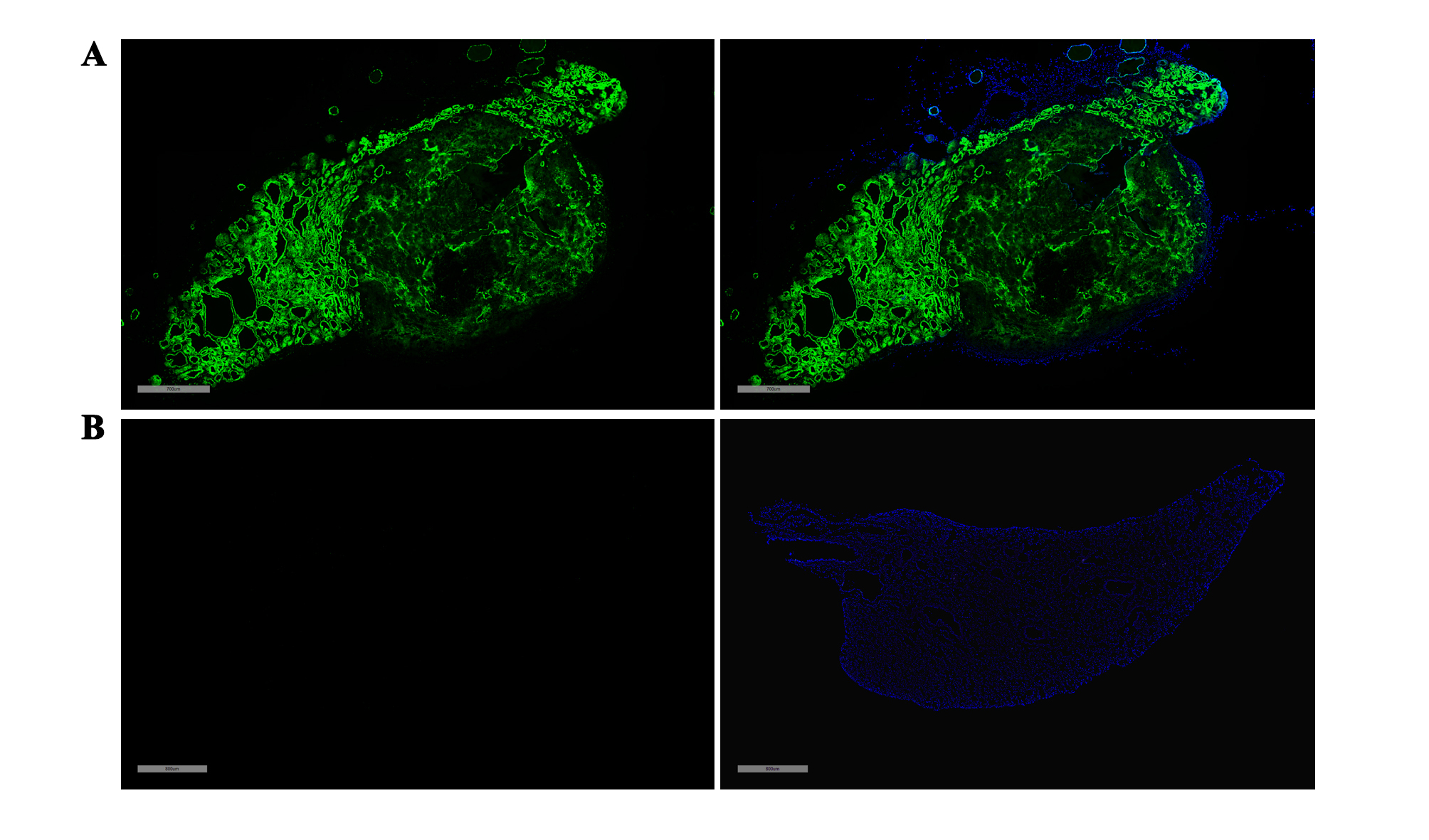

Supplement: Supplementary file 2 — Additional file 2. Figure S2: Exclusion on non-specific activation of MMTV in the lungs. The lung of 18-week-old MPGor MMTV-Greenmice were collected and detected by fluorescent microscope. Scale bar, 700 μm. [file 13058_2023_1662_MOESM2_ESM.jpg]

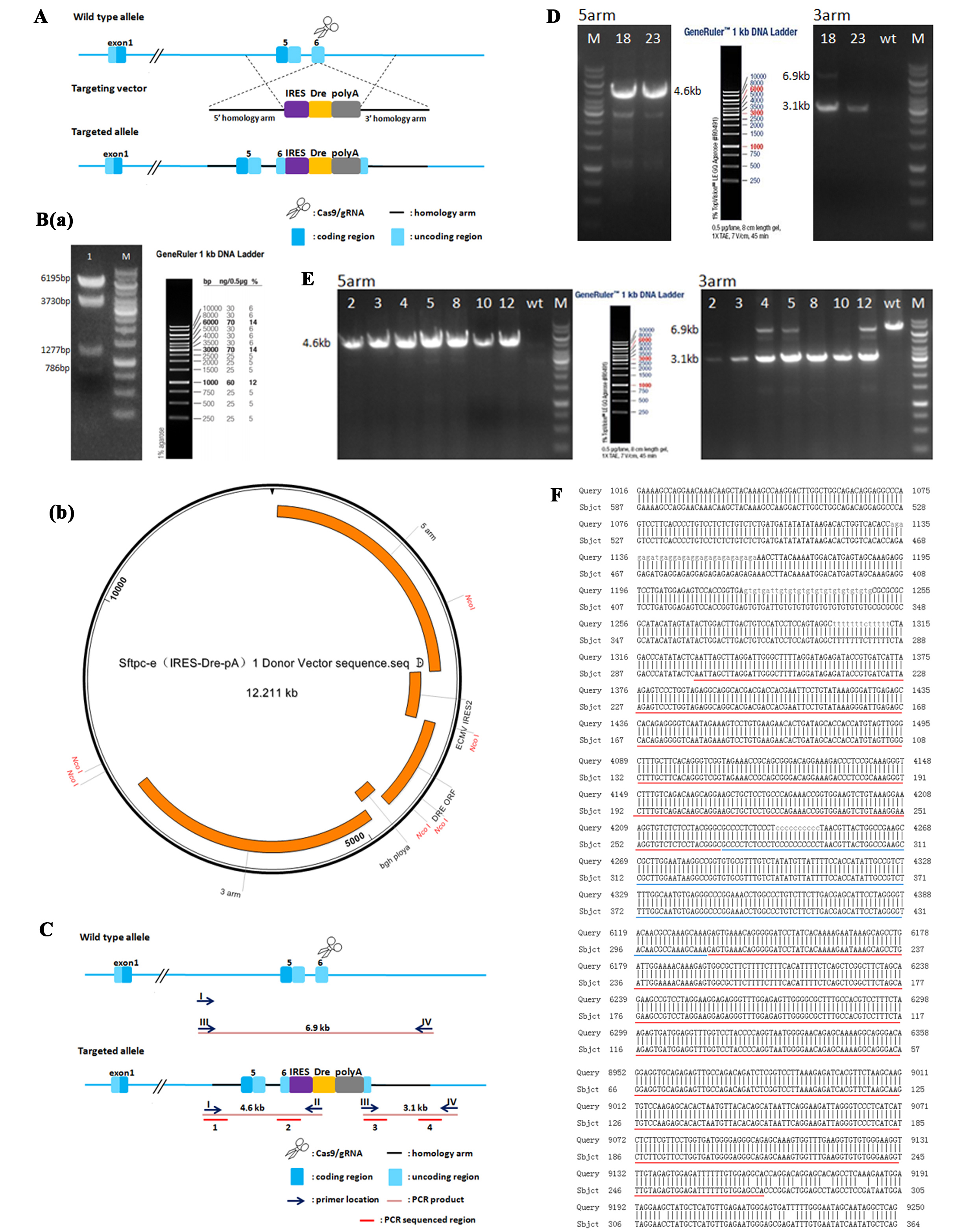

Supplement: Supplementary file 3 — Additional file 3. Figure S3: Construction of Sftpc-Dre mouse. A Schematic diagram of design strategy. CRISPR/cas9 technology was used to knock in the IRES-Dre-pA expression boxat exon 6 site of Sftpc gene by homologous recombination. B Constructionand identificationof the donor vector. Cas9 mRNA and gRNA were obtained by in vitro transcription. The In-Fusion cloning method was used to construct the donor vector, which contained 2.9 kb 5’ homologous arm, IRES-Dre-pA and 3.0 kb 3’ homologous arm. C Identification strategy of F0 generation mice. The F0 generation mice were obtained by microinjection of Cas9 mRNA, gRNA and donor vector into the fertilized eggs of C57BL/6J mice. The 5’ homologous recombination-positive genome should amplify the 4.6-kb fragment, whereas the negative genome has no fragment; the 3.1-kb fragment should be amplified from the 3’ homologous recombination-positive genome, and a 6.9-kb fragment should be amplified from the negative genome. D PCR identification results of positive F0 generation mice. The F0 generation mice with positive double arm homologous recombination were No.18 and No. 23. Number, F0 mouse number; WT, wild-type control; M, 1 kb DNA marker. E The 5' and 3' homologous arms of F1 generation mice were identified by PCR. F0 generation mice are chimeric; they do not necessarily have the ability of stable inheritance. Therefore, these F0 generation mice need to be subcultured to obtain stable F1 generation mice. F0 generation positive mice were mated with wild-type C57BL/6J mice to obtain F1 generation mice. F The alignment between the target sequences and the PCR products of the F1 generation mice. Query, target sequence1 recombinant genomic DNA sequence); Subject, sequencing result of PCR products; Red underline, the 5’ or 3’ homologous arm sequence; Blue underline, knock in sequence. [file 13058_2023_1662_MOESM3_ESM.jpg]

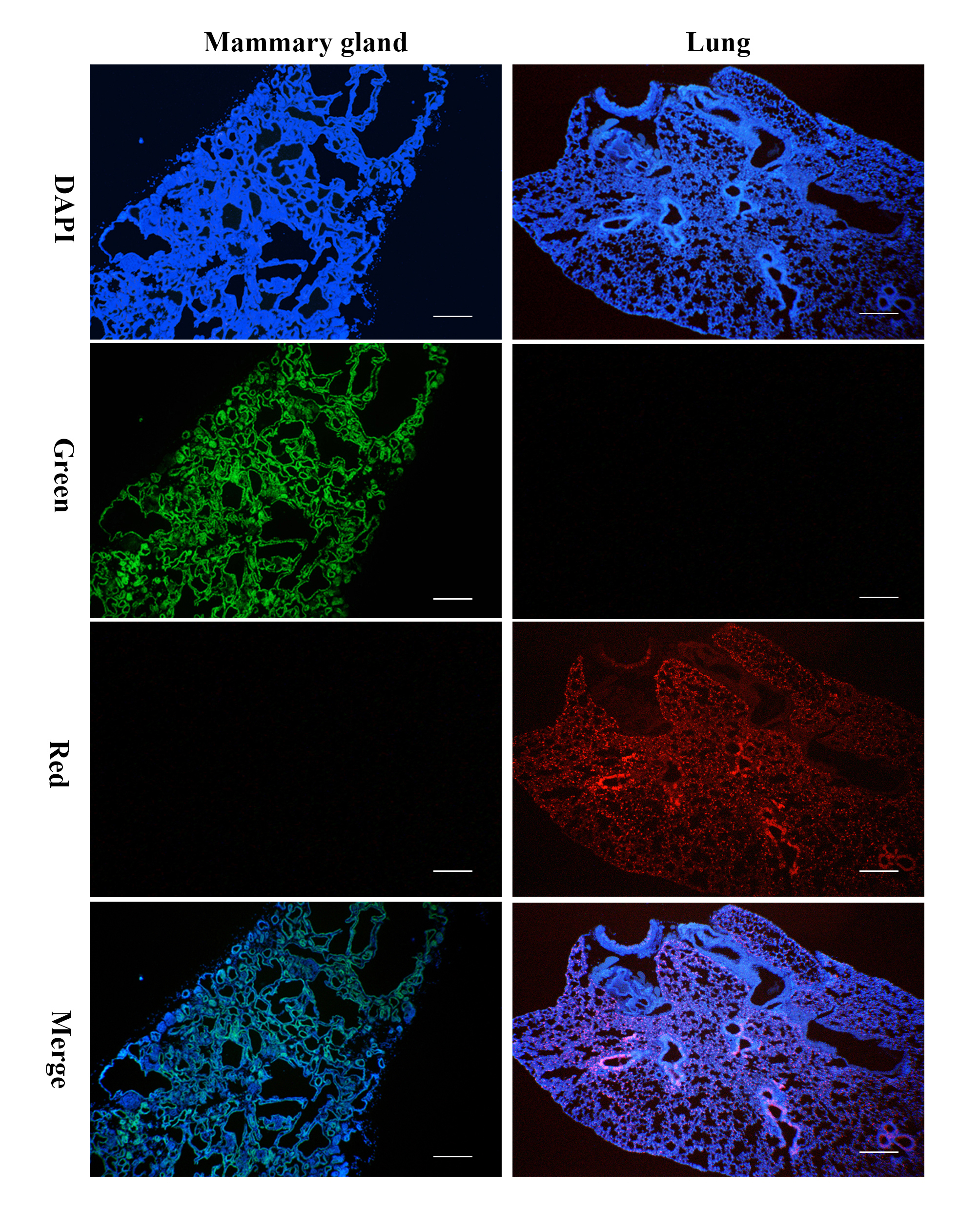

Supplement: Supplementary file 4 — Additional file 4. Figure S4: Label mice with two recombination systems. The mammary gland and lung of 3-week-old MPG-ST mice were collected and cut into slices for detection under fluorescence microscope. Representative images were showed. n = number of mice. Scale bar, 320 μm. [file 13058_2023_1662_MOESM4_ESM.jpg]

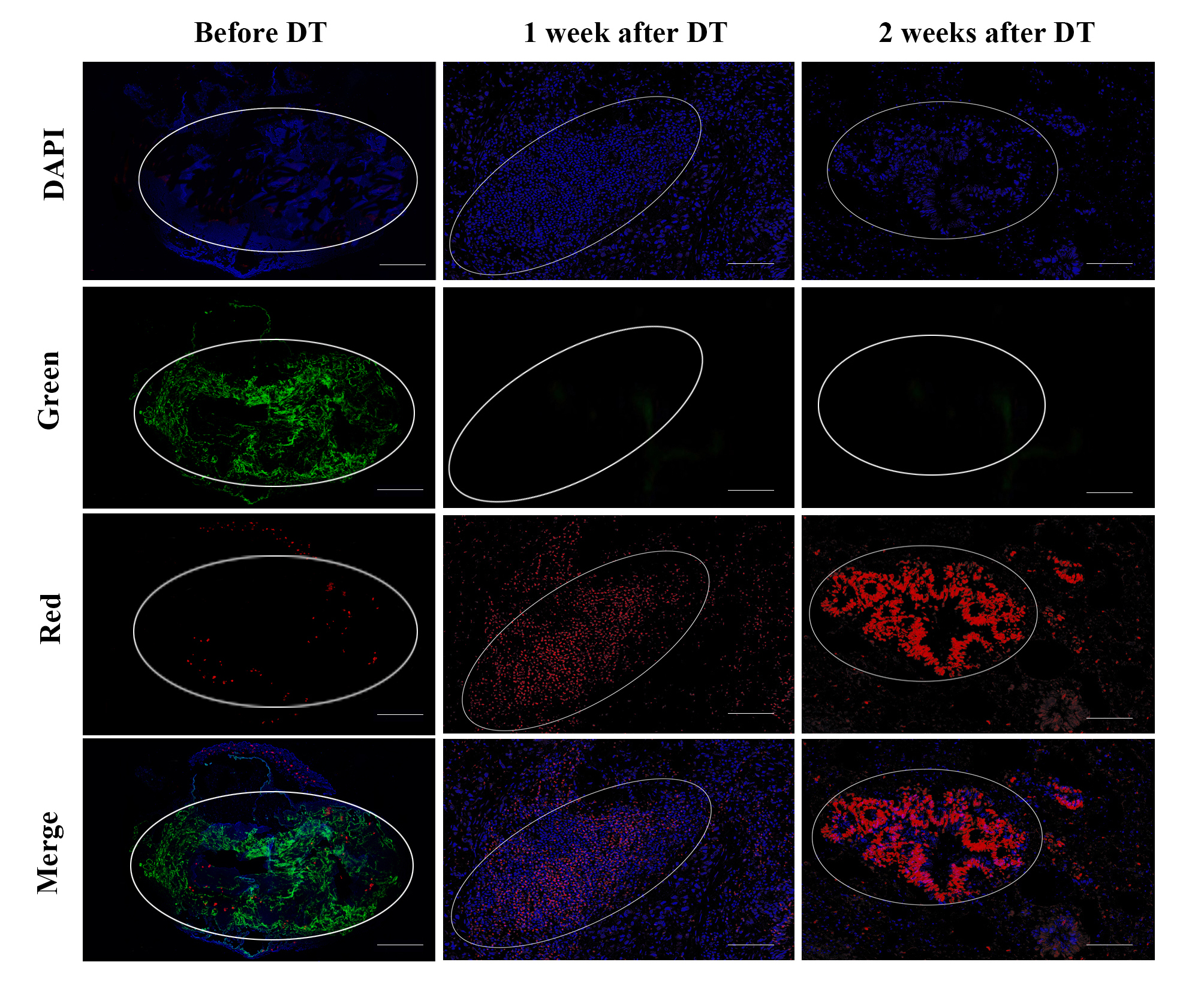

Supplement: Supplementary file 6 — Additional file 6. Figure S5: The regenerative growth of metastasis after DT injection. MPG-ST-iDTR miceat 16 weeks were injected with 100 ng diphtheria toxinevery 8 h for 7 consecutive days. Lung metastases were detected by fluorescence microscope. Representative images of lung sections without DT treatmentand at 1 weekor 2 weeksafter DT injection. Circle mark indicate the tumor site. n = number of mice groups. Scale bar, 320 μm [file 13058_2023_1662_MOESM6_ESM.jpg]

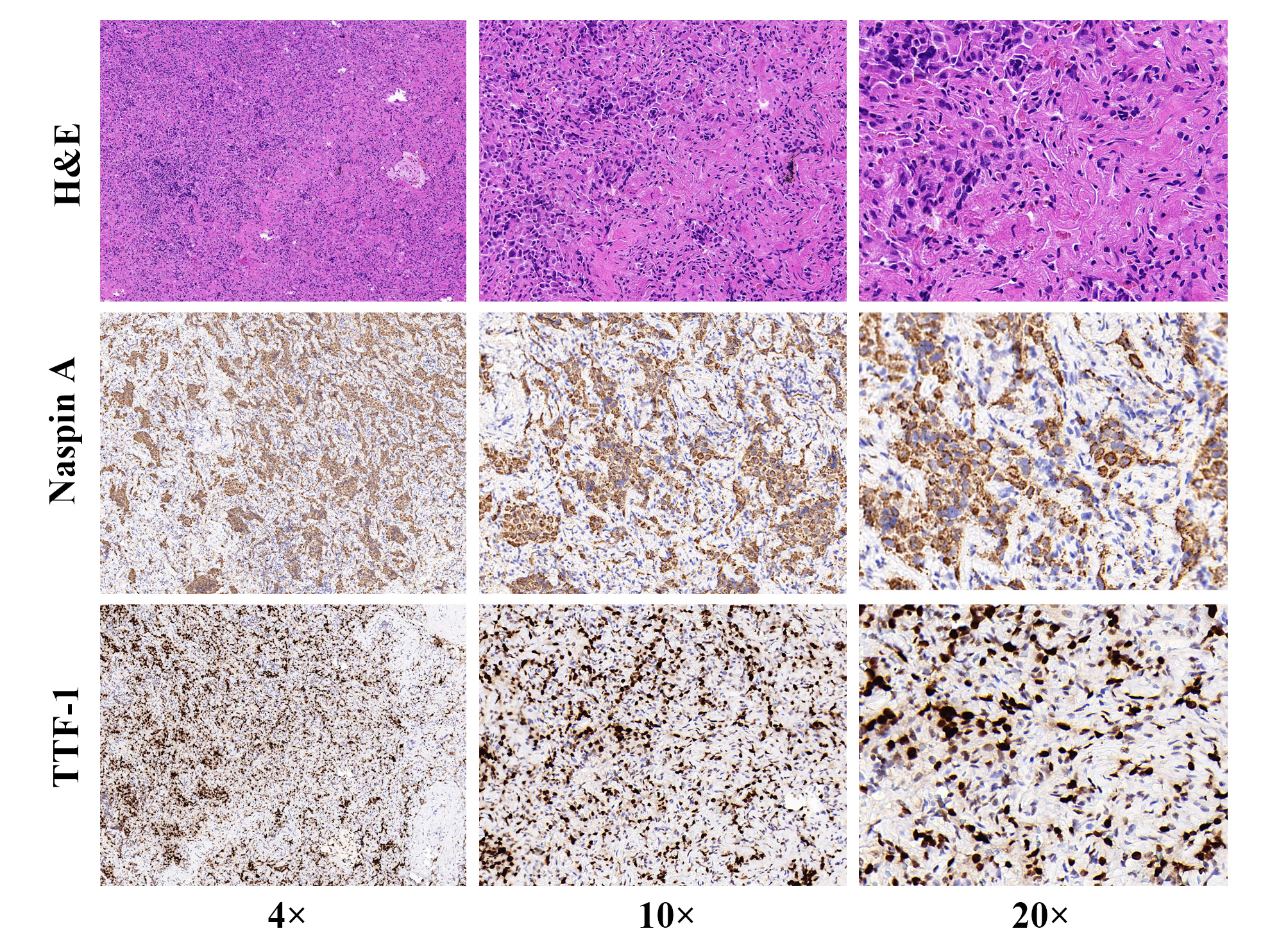

Supplement: Supplementary file 7 — Additional file 7. Figure S6: AT2 cells from the new generated tumor are malignant. MPG-ST-iDTR miceat 16 weeks were injected with 100 ng diphtheria toxinevery 8 h for 7 consecutive days. After DT injection for 2 weeks, AT2 cells among the lung metastasis were FACS-sorted and subcutaneously transplantedinto syngeneic wild-type mice. H&E and immunohistochemistry for adenocarcinoma markerswere applied after growth for 18 weeks. Magnification, 4×, 10×and 20×. n=number of mice. [file 13058_2023_1662_MOESM7_ESM.jpg]

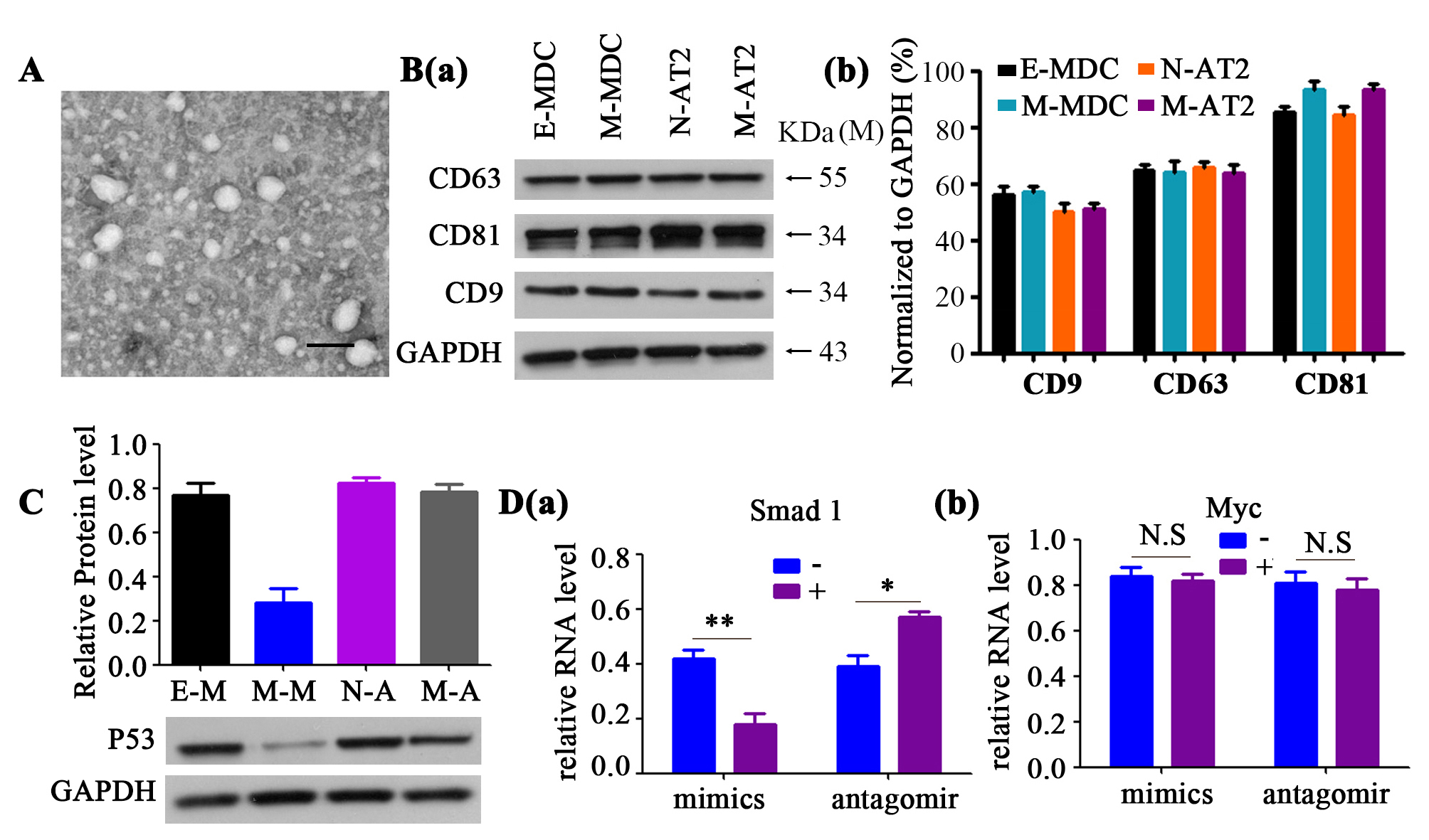

Supplement: Supplementary file 9 — Additional file 9. Figure S7: PCR array and exosomes detection. A Transmission electron microscopy of exosomes secreted by malignant MDCs. Scale bar, 100 nm. B Classic exosome biomarkers were detected by western blotting. Quantification of relative protein level. C E-MDC, M-MDC, N-AT2and M-AT2cellswere collected. Their P53 protein expression were detected by western blotting. Data were quantified and presented as the mean ± SD of triplicate experiments. D The tdTomato-positive cellswere FACS sorted from the lung metastasis of the MPG-ST-iDTR mice 2 weeks after DT treatment and transfected with miR-675 mimics or antagomir. The RNA expression of Smad 1and Mycwas measured and quantifiedby qRT-PCR. Data presented are the mean of triplicate experiments. Error bars indicate standard deviation. N.S, no significance; *, p<0.05; **, p<0.01. [file 13058_2023_1662_MOESM9_ESM.jpg]

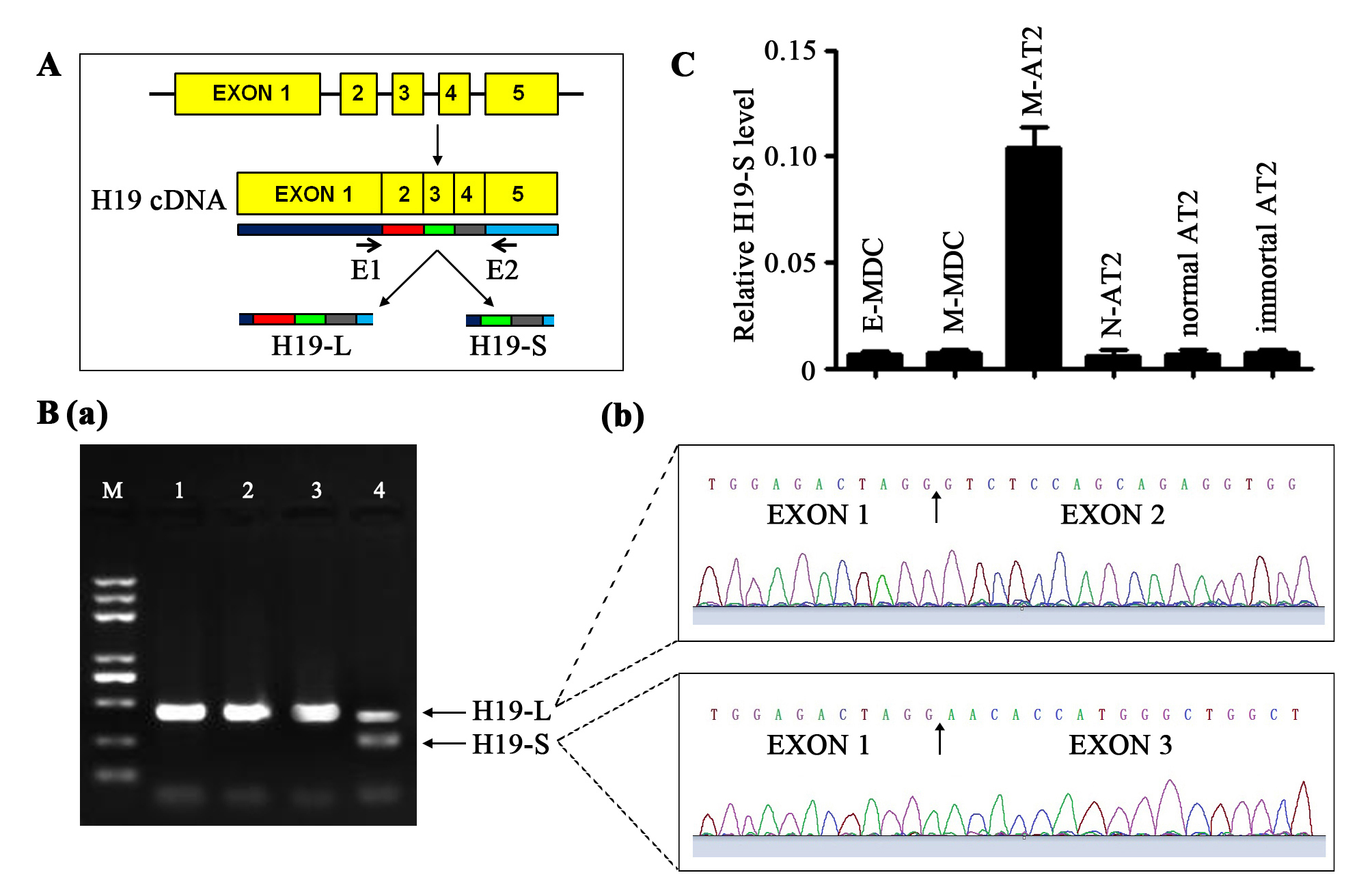

Supplement: Supplementary file 12 — Additional file 12. Figure S8: Alternative splicing of H19 lncRNA in malignant AT2 cells. A Schemes illustrating PCR amplification of H19 variants. E1 and E2 indicate PCR primers for H19 cDNA. B RT-PCRand sequenceanalysis for H19 transcript using E1 and E2 primers. M, DNA ladder; Lane 1: E-MDCs; Lane 2: M-MDCs; Lane 3: N-AT2 cells; Lane 4: M-AT2 cells. C The RNA level of H19 without exon 2 in indicated AT2 cells were analyzed by qRT-PCR and normalized to U6 snRNA. Data presented are the mean of triplicate experiments. Error bars indicate standard deviation. H19-L, full length H19; H19-S, H19 without exon 2. Abbreviations: MDCs, mammary gland-derived cells; E-MDCs, early MDCs; M-MDCs, malignant MDCs; N-AT2 cells, normal AT2 cells; M-AT2 cells, malignant AT2 cells. [file 13058_2023_1662_MOESM12_ESM.jpg]

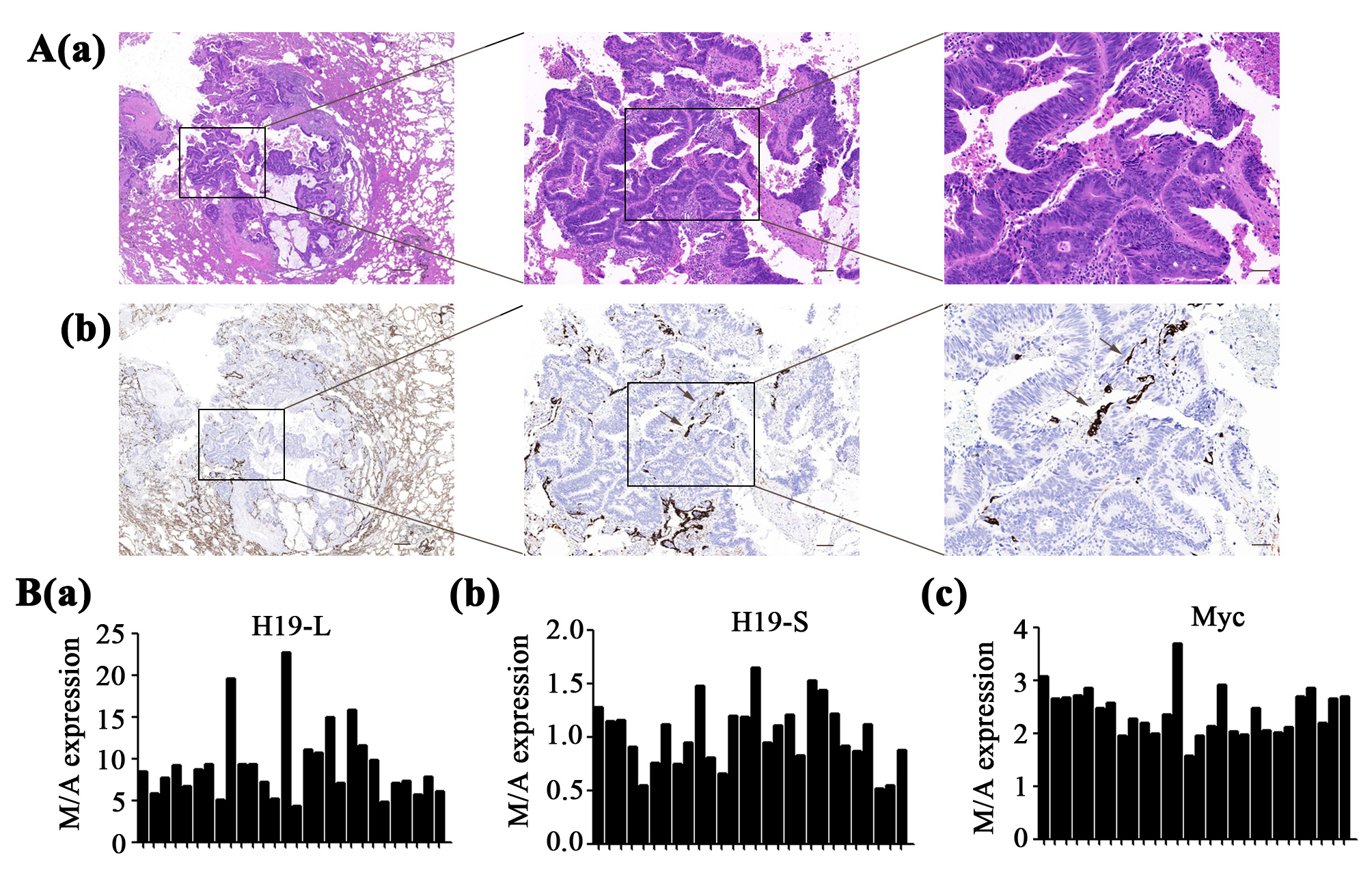

Supplement: Supplementary file 13 — Additional file 13. Figure S9: Investigation in clinical samples. A H&Eor IHCfor Sftpc were applied onto the slices of lung metastasis from MBC patients. Arrows show the Sftpc positive cells. left panel: scar bar, 320um; magnification, 1×. middle panel: scar bar, 80um; magnification, 4×. right panel: scar bar, 40um; magnification, 10×. B The RNA expression of H19-L, H19-S or Myc in lung metastasesand their paired adjacent tissuesof 28 MBC patients were analyzed by qRT-PCR. The results were presented by M/A expression and normalized to U6 snRNA. [file 13058_2023_1662_MOESM13_ESM.jpg]

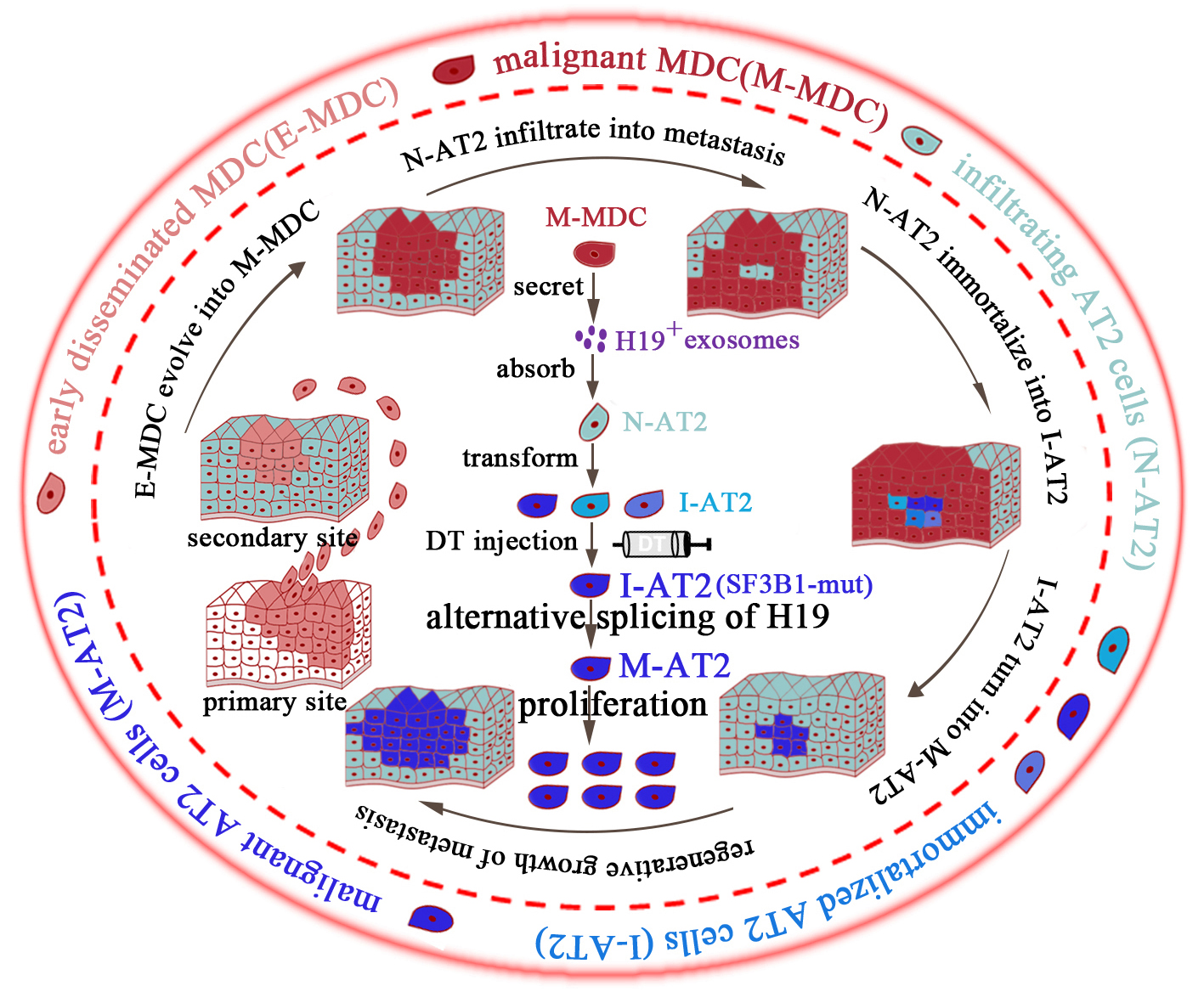

Supplement: Supplementary file 14 — Additional file 14. Figure S10: Summary of the regenerative growth of metastasis. Schematic diagram of metastasis and regenerative growth of metastasis. [file 13058_2023_1662_MOESM14_ESM.jpg]
