# Supplementary material for: Breast metastatic tumors in lung can be substituted by lung-derived malignant cells transformed by alternative splicing H19 lncRNA
Source: Breast Cancer Res. 2023 May 30;25:59. doi: 10.1186/s13058-023-01662-z (PMC10228081; doi:10.1186/s13058-023-01662-z)
Supplement: Supplementary file 5 — Additional file 5. Table S1: Lung metastasis after DT treatment [file 13058_2023_1662_MOESM5_ESM.pdf]

Table S1 Lung metastasis after DT treatment

Time point (DT injection)	Time (after DT injection)	Number (mice)	Number (lung metastasis)
12 weeks old	0 week	10	2.80±0.79
	1 week	10	0
	2 weeks	10	0
14 weeks old	0 week	10	4.90±0.74
	1 week	10	0
	2 weeks	10	0
16 weeks old	0 week	10	8.60±0.96
	1 week	10	4.90±1.19
	2 weeks	9	6.44±1.25
18 weeks old	0 week	10	13.1±1.20
	1 week	8	8.75±1.28
	2 weeks	6	10±1.89
