# Supplementary material for: Breast metastatic tumors in lung can be substituted by lung-derived malignant cells transformed by alternative splicing H19 lncRNA
Source: Breast Cancer Res. 2023 May 30;25:59. doi: 10.1186/s13058-023-01662-z (PMC10228081; doi:10.1186/s13058-023-01662-z)
Supplement: Supplementary file 11 — Additional file 11. Table S2: The highly expressed LncRNAs in the exosomes from M-MDC cells. [file 13058_2023_1662_MOESM11_ESM.pdf]

Table S2 The highly expressed LncRNAs in the exosomes from M-MDC cells

LncRNA	Fold change	P-value	FDR
FR0257520	63.19984367	0.000455424	0.000154841
FR0201871	27.27374206	0.00325061	0.000234135
FR0402396	22.57137273	0.000734269	1.22169E-05
FR0041840	19.74995116	0.00160044	6.99331E-05
FR0407528	17.86900342	0.0042033	0.00031761
FR0241205	16.92852955	0.00607522	0.000807964
FR0378527|H19	12.51786756	0.00164701	0.000950862
FR0059284	8.06120455	0.000776735	0.000316686
FR0212673	6.348198585	0.00149182	0.00060579
FR0186572	6.191452939	0.00289472	0.000838163
FR0019280	5.980449189	0.00242636	0.000939276
FR0028402	5.642843188	0.00458618	0.000190211
FR0185138	4.937487786	0.00166215	0.000414024
FR0203919	3.174099291	0.0088231	0.000419745
FR0328380	3.117215792	0.00236524	0.000240157
FR0299679	2.945635121	0.0017727	0.000245125
FR0344267	2.821421592	0.00581308	0.000224506
FR0365848	2.477345788	0.00524869	0.000128837
FR0265053	2.257137274	0.00160205	0.000136848
FR0154071	2.116066194	0.00385161	0.000589374
