# Supplementary material for: Breast metastatic tumors in lung can be substituted by lung-derived malignant cells transformed by alternative splicing H19 lncRNA
Source: Breast Cancer Res. 2023 May 30;25:59. doi: 10.1186/s13058-023-01662-z (PMC10228081; doi:10.1186/s13058-023-01662-z)
Supplement: Supplementary file 15 — Additional file 15. Data file S3: Sequences and primers. [file 13058_2023_1662_MOESM15_ESM.doc]

**Data file S2-Sequences and primers**

**>mmu-let-7a-2**

CUGUACAGCCUCCUAGCUUUC

**>Mus musculus H19 NR_130974.1 ( H19-L )**

accgggtgtgggaggggggtggggggtgggggtggggggtatcggggaaactggggaagatgggagagctggaggagagtcgtggggtccgaggagcacctcggcatctggagtctggcaggaatgttgaaggactgaggggctagctcaggcagagcaaaggcatcgcaaaggctggaaaacatcggagtgaagctgaagggcctgagctagggttggagaggaatggggagccagacattcatcccggttacttttggttacaggacgtggcggctggtcggataaaggggagctgctgggaagggttcgaccccagacctgggcagtgaaggtatagctggcagcagtgggcaggtgaggaccgccgtctgctgggcaggtgagtctccttcttctctcttggcctcgctccactgaccttctaaacgaaggtttagagagggggcctggtgagaagaagcggctggcctcgcagcagaatggcacatagaaaggcaggatagttagcaaaggagacatcgtctcggggggagccgagacagaaggaggctgggggaccattggcgaccccaggtggaaagagctcttagagagaagaaagaagaggtgcagggttgccagtaaagactgaggccgctgcctccagggaggtgataggagtccttggagacagtggcagagaccatgggatccagcaagaacagaagcattctaggctggggtcaaacagggcaagatggggtcacaagacacagatgggtccccagccgccacaacatcccacccaccgtaattcacttagaagaaggttcaagagtggctctggcaaagtcccaagtttgccagagcctcaataactggagaatggaaaagaagggcagtgcagggtgtcaccagaaggggagtgggggctgcaggtatcggactccagagggattttacagcaaggaggctgcagtgggtccagcctgcagacacaccattcccatgaggcactgcggcccagggactggtgcggaaagggcccacagtggacttggtacactgtatgccctaaccgctcagtccctgggtctggcatgacagacagaacatttccaggggagtcaagggcacaggatgaagccagacgaggcgaggcaggcggggcagaatgaatgagtttctagggagggaggttgggtgcaggtagagcgagtagctggggtggtgagccagggaggcactggcctccagagtccgtggccaaggagggccttgcgggcggcgacggagcagtgatcggtgtctcgaagagctcggactggagactagggtctccagcagaggtggatgtgcctgccagtcactgaaggcgaggatgacaggtgtggtcaatgtgacagaaagacatgacatggtccggtgtgatggagaggacagaagggcagtcatccagccttcttgaacaccatgggctggcgccttgtcgtagaagccgtctgttctttcacttttcccaaagagctaacacttctctgctgctctctggatcctcctccccctaccttgaaccctcaagatgaaagaaatggtgctacccagctcatgtctgggcctttgaatccggggacttctttaagtccgtctcgttctgaatcaagaagatgctgcaatcagaaccactacactacctgcctcaggaatctgctccaaggtgaagctgaaagaacagatggtgtcaacattttgaaagagcagactcatagcacccacccacccctgagaatccatcttcatggccaactctgcctgacccgggagaccaccacccacatcatcctggagccaagcctctaccccgggatgacttcatcatctccctcctgtctttttcttcttcctcctttcctgtaattctgtttctttccttttgttccttccttgcttgagagactcaaagcacccgtgactctgtttccccatttacccccttttgaatttgcactaagtcgattgcactggtttggagtcccggagatagctttgagtctctccgtatgaatgtatacagcgagtgtgtaaacctctttggcaatgctgccccagtacccacctgtcgtccatctccgtctgagggcaactgggtgtggccgtgtgcttgaggcctcgccttcccctcgcctagtctggaagcagttccatcataaagtgttcaacatgccctacttcatcctttgcccctcctcaccagggcctcaccagaggtcctgggtccatcaataaatacagttacagtcat

**H19 Without exon 2 ( H19-S )**

accgggtgtgggaggggggtggggggtgggggtggggggtatcggggaaactggggaagatgggagagctggaggagagtcgtggggtccgaggagcacctcggcatctggagtctggcaggaatgttgaaggactgaggggctagctcaggcagagcaaaggcatcgcaaaggctggaaaacatcggagtgaagctgaagggcctgagctagggttggagaggaatggggagccagacattcatcccggttacttttggttacaggacgtggcggctggtcggataaaggggagctgctgggaagggttcgaccccagacctgggcagtgaaggtatagctggcagcagtgggcaggtgaggaccgccgtctgctgggcaggtgagtctccttcttctctcttggcctcgctccactgaccttctaaacgaaggtttagagagggggcctggtgagaagaagcggctggcctcgcagcagaatggcacatagaaaggcaggatagttagcaaaggagacatcgtctcggggggagccgagacagaaggaggctgggggaccattggcgaccccaggtggaaagagctcttagagagaagaaagaagaggtgcagggttgccagtaaagactgaggccgctgcctccagggaggtgataggagtccttggagacagtggcagagaccatgggatccagcaagaacagaagcattctaggctggggtcaaacagggcaagatggggtcacaagacacagatgggtccccagccgccacaacatcccacccaccgtaattcacttagaagaaggttcaagagtggctctggcaaagtcccaagtttgccagagcctcaataactggagaatggaaaagaagggcagtgcagggtgtcaccagaaggggagtgggggctgcaggtatcggactccagagggattttacagcaaggaggctgcagtgggtccagcctgcagacacaccattcccatgaggcactgcggcccagggactggtgcggaaagggcccacagtggacttggtacactgtatgccctaaccgctcagtccctgggtctggcatgacagacagaacatttccaggggagtcaagggcacaggatgaagccagacgaggcgaggcaggcggggcagaatgaatgagtttctagggagggaggttgggtgcaggtagagcgagtagctggggtggtgagccagggaggcactggcctccagagtccgtggccaaggagggccttgcgggcggcgacggagcagtgatcggtgtctcgaagagctcggactggagactaggaccatgggctggcgccttgtcgtagaagccgtctgttctttcacttttcccaaagagctaacacttctctgctgctctctggatcctcctccccctaccttgaaccctcaagatgaaagaaatggtgctacccagctcatgtctgggcctttgaatccggggacttctttaagtccgtctcgttctgaatcaagaagatgctgcaatcagaaccactacactacctgcctcaggaatctgctccaaggtgaagctgaaagaacagatggtgtcaacattttgaaagagcagactcatagcacccacccacccctgagaatccatcttcatggccaactctgcctgacccgggagaccaccacccacatcatcctggagccaagcctctaccccgggatgacttcatcatctccctcctgtctttttcttcttcctcctttcctgtaattctgtttctttccttttgttccttccttgcttgagagactcaaagcacccgtgactctgtttccccatttacccccttttgaatttgcactaagtcgattgcactggtttggagtcccggagatagctttgagtctctccgtatgaatgtatacagcgagtgtgtaaacctctttggcaatgctgccccagtacccacctgtcgtccatctccgtctgagggcaactgggtgtggccgtgtgcttgaggcctcgccttcccctcgcctagtctggaagcagttccatcataaagtgttcaacatgccctacttcatcctttgcccctcctcaccagggcctcaccagaggtcctgggtccatcaataaatacagttacagtcat

**3'UTR clone of Myc**

CAATTGGCAGAGCTCAGAATTCAAGCGATCGCCTACGGAACTCTTGTGCGTAAGGAA

AAGTAAGGAAAACGATTCCTTCTAACAGAAATGTCCTGAGCAATCACCTATGAACTT

GTTTCAAATGCATGATCAAATGCAACCTCACAACCTTGGCTGAGTCTTGAGACTGAA

AGATTTAGCCATAATGTAAACTGCCTCAAATTGGACTTTGGGCATAAAAGAACTTTT

TTATGCTTACCATCTTTTTTTTTTCTTTAACAGATTTGTATTTAAGAATTGTTTTTA

AAAAATTTTAAGATTTACACAATGTTTCTCTGTAAATATTGCCATTAAATGTAAATA

ACTTTAATAAAACGTTTATAGCAGTTACACAGAATTTCAATCCTAGTATATAGTACC

TAGTATTATAGGTACTATAAACCCTAATTTTTTTTATTTAAGTACATTTTGCTTTTT

AAAGTTGATTTTTTTCTATTGTTTTTAGAAAAAATAAAATAACTGGCAAATATATCA

TTGAGCACGCGTAAGCGGCCGCGGCATCTAGATTCGAAGAAAATGACCG

**Primers**

*Primers for H19 variants*

H19, E1,5’-AGCAGTGATCGGTGTCTCGAAGA

H19, E2,5’-AGTCTGCTCTTTCAAAATGTTGAC

*Primers for H19 clones*

pH19-L, F,5’-TGGCCCACACCCTCCCCCCACAAGCTT

pH19-L, R,5’-ATGACTGTAACTGTATTTATTGATGGCTGGAG

pH19-S, F,5’-CCATGGTCCTAGTCTCCAGTCCGA

pH19-S, R,5’-TGGAGACTAGGACCATGGGCTGGCGC

pH19-mut, F,5’-CCATGGTCGTAGTTTCCAGTCCGA

pH19-mut, R,5’-TGGAAACTACGACCATGGGCTGGCGC

SF3B1-WT, F,5’-GGATCCTACCGCTTCTAGCGGTTCTGAG

SF3B1-WT, R,5’-GATATCGACATATTCATCCATGTTATCTAT

SF3B1-mut, F,5’-ACTTATACAGGCTTTGAGTTGTCGAT

SF3B1-mut, R,5’-TCCATGTTATCTATATCAGGTCTC

*Primers for qRT-PCR*

H19-L, F,5’-TGGGGTGGTGAGCCAGGGAGGC

H19-L, R,5’-TCTCCATCACACCGGACCAT

H19-S, F,5’-GGAGCAGTGATCGGTGTCTCGA

H19-S, R,5’-AGGATCCAGAGAGCAGCAGAGA

Myc, F,5’-AGCCACGACGATGCCCCTCAACG

Myc, R,5’-TGTGTGTTGCAGAACCTTGCAGT

**Oligonucleotides**

*pSiCHECK2-let-7a 4*×

GAACCACTACACTACCTGCCTCAGGCGGCTCGAGGAACCACTACACTACCTGCCTCA

GGAACCACTACACTACCTGCCTCAGGAACCACTACACTACCTGCCTCAGGAACCACT

ACACTACCTGCCTCAGGCGGCCGCAA

**IRES-Dre-pA**

TTCTCATGTTTGACAGCTTATCATCGATAAGCTGCGGCCGCAAAGGCCGCGGTCGACAAGCTaattagcttaggattgggcttttaggatagagataccgtgatcattaagagtccctggtagaggcaggcacgacgaccacgaattcctgtataaagggattgagagccacagaggggtcaatagaaagtcctgtgaagaacactgatagcaccaccatgtagttgggaggctacaggaagtaccacagagcaaatgacagtgaggctgggatgtagatcagtagctagagcactttgcaaacgtggagacctgggttcagtccccagtaccacataatccaggcatagaggtgcacactgtttggaaatataagcaagataatcattcagggtcatccttagatatataaggagttgaaaggcagcctgggtcatagatatggtttttttctttaaaaaaaaaaaaaggatggctcagtaggtagtggtgcttgccaccaaatgtggcggctcgagtttaatctctggaattcacatgatgaaaggagatgaaagttgtccctgacttacacacacacatgtacatacacactcacacatactacacacatgtacacaaacattcacactcacaccacatacacacactctcacacacaccacacacatgtgcacacacacacacacacatgtaccttcacacacatcacacacatgcacactcaaacataccacacacatgtatacacgctcacacactaaataagtgtaaaaaaccatgaagctggcacatggactggattgagaaggaacaaaggagacccgtgtaaaggtatagagacagggctacctggatcctttgagggagcaaaaactcaggatatgtctggggtgttaggcgtcgtgggaaaacagaagaagagatcggtagctggctctcttggattatttcaaacctagactttgcttctaactttctaagtagctatggttttgttagggtccaaccaccttccccaaagccttccttcccaaaagcctctgatctccccagctcttccttgcacactcgtagcctaaagaggtacgggatgtgtgtgtcccccttgcacagggagagtaggagccaccctctccactacccacttgtctctctgcttcccttgctctctcagGATTACTCGGCAGGTCCCAGGAGCCAGTTCCGCATCCCCTGCTGTCCCGTGCACCTCAAACGCCTTCTCATCGTGGTTGTGGTGGTGGTCCTCGTTGTCGTGGTGATTGTAGGGGCTCTGCTCATGGGCCTCCACATGAGTCAAAAACATACTGAGATGgtgagtgggcctgggttgggcaaagaggcacagcagacagggggttgggggagattatggggggatgggcagctgttcgggaggaagagaagggagtggacaggtatgagcacatcttgggtgacacaaacagagacgaggtagccatcctgcctagatcctctcccccaggccccggcctagtgtgataaccatcgattgctctgacacctcttacgtttagccttcctgagatctcaggaaagcgtttgaatagaggattctgagtagatatgggtaccgaaaggctgagggaagaaagagaaataccaggcagcattccaacacccttcccctagGTCCTTGAGATGAGCATCGGAGCACCGGAAACTCAGAAACGCCTAGCCCCGAGTGAGCGAGCAGACACCATCGCTACCTTTTCCATCGGCTCCACTGGCATCGTTGTGTATGACTACCAGCGGgtgaggatgccggagggaccaccgggactttattggaactagccagttgtagcatttctagaggtctctccccattctgtgcctggctacctcacctcagatgctcgaaccactgacgcaagtgcgcccctccaccctctgaagacaatctaaaggaagttggttggctgagaactagggttggggaggaagcaaggcaaggggaccttgtgaatgacctccagggttttatacctagCTCCTGACGGCCTATAAGCCAGCTCCAGGAACCTACTGCTACATCATGAAGATGGCTCCAGAGAGCATCCCTAGTCTTGAGGCTTTCGCTAGAAAACTCCAGAACTTCCAGgtgggtatgttagggagggagggagcagtctccctctgagggttgagtagagggacatgtgaaaggatgactagcgtaccctgtgtagtattgatgtttctggatcagacatgttctcctctctccatggacctgtgtcctgccatccctaccagctctcaggtggccctgctaagttgttggccttggctgagcttagacatgacctatgggcttctacatccaacccagtccctctctgaatttgtgaggaaactgatcctcgagaattaccaacttagtgtcccacactaaataaagcaggtgacattgaaagtaggtgttctttccagGCCAAGCCCTCCACACCCACCTCTAAGCTGGGCCAGGAGGAAGGGCATGATACTGGTTCCGAGTCCGATTCTTCCGGGAGAGACCTGGCTTTCCTAGGCCTTGCTGTGAGCACCCTGTGTGGAGAGCTACCACTCTACTATATCTAGCATCCACAGgtgagcaacagtacctttcagggtgcctgggcaacactggcagggcttgggctgcctgctttgtcaggggacctactaggtatctcttaaagtcagtggtctcgggagctcggaggatggagggttcccagacacatcccacactggacccaagcgggtggcttcttcagtccccataagcattagttctttgcttcacagGGTCGGTAGAAACCGCAGCGGGACAGGAAAGACCCTCCGCAAAGGGTCTTTGTCAGACAAGCAGGAAGCTGCTCCTGCCCAGAAACCGGTGGAAGTCTGTAAAGGAAAGGTGTCTCTCCTACGGGCGCCCCTCTCCCTCCCCCCCCCCTAACGTTACTGGCCGAAGCCGCTTGGAATAAGGCCGGTGTGCGTTTGTCTATATGTTATTTTCCACCATATTGCCGTCTTTTGGCAATGTGAGGGCCCGGAAACCTGGCCCTGTCTTCTTGACGAGCATTCCTAGGGGTCTTTCCCCTCTCGCCAAAGGAATGCAAGGTCTGTTGAATGTCGTGAAGGAAGCAGTTCCTCTGGAAGCTTCTTGAAGACAAACAACGTCTGTAGCGACCCTTTGCAGGCAGCGGAACCCCCCACCTGGCGACAGGTGCCTCTGCGGCCAAAAGCCACGTGTATAAGATACACCTGCAAAGGCGGCACAACCCCAGTGCCACGTTGTGAGTTGGATAGTTGTGGAAAGAGTCAAATGGCTCTCCTCAAGCGTATTCAACAAGGGGCTGAAGGATGCCCAGAAGGTACCCCATTGTATGGGATCTGATCTGGGGCCTCGGTGCACATGCTTTACATGTGTTTAGTCGAGGTTAAAAAAACGTCTAGGCCCCCCGAACCACGGGGACGTGGTTTTCCTTTGAAAAACACGATGATAATATGGCCACAACCATGGGTGCTAGCGAGCTGATCATCTCTGGCTCCTCTGGAGGATTCCTGAGGAACATCGGCAAGGAGTACCAGGAGGCTGCTGAGAACTTCATGAGATTCATGAATGACCAGGGAGCCTACGCCCCTAACACCCTGAGAGACCTGAGGCTGGTGTTCCACTCCTGGGCTAGATGGTGCCACGCTAGACAGCTGGCCTGGTTCCCTATCTCTCCTGAGATGGCTAGGGAGTACTTCCTTCAGCTGCACGATGCTGACCTGGCCTCTACCACCATCGACAAGCACTACGCCATGCTGAACATGCTGCTGTCCCACTGTGGCCTGCCTCCTCTGTCTGATGACAAGTCTGTGAGCCTGGCCATGAGGAGAATCCGGAGAGAGGCTGCCACCGAGAAGGGAGAGAGAACCGGCCAGGCCATCCCTCTGAGATGGGATGACCTGAAGCTGCTGGATGTGCTGCTGTCTAGATCTGAGAGACTGGTGGACCTGAGGAATAGGGCCTTCCTGTTTGTGGCCTACAACACCCTGATGAGGATGTCTGAGATCTCTAGGATCAGAGTGGGAGACCTGGACCAGACCGGAGACACCGTGACCCTGCACATCTCCCACACCAAGACCATCACCACCGCTGCTGGCCTGGACAAAGTGCTGTCTAGGAGGACCACCGCTGTGCTGAATGACTGGCTGGATGTGTCTGGCCTGAGAGAGCACCCTGACGCTGTGCTGTTCCCTCCTATCCACCGGAGCAACAAGGCTAGGATCACCACCACCCCTCTGACCGCCCCTGCCATGGAGAAGATTTTTAGCGATGCCTGGGTGCTGCTGAACAAGAGGGATGCCACCCCTAACAAGGGCCGCTACCGGACCTGGACCGGCCACTCTGCTAGAGTGGGAGCTGCCATCGACATGGCTGAGAAGCAAGTGTCCATGGTGGAGATCATGCAGGAGGGCACCTGGAAAAAGCCTGAGACACTGATGAGATACCTGAGGAGGGGAGGAGTGTCTGTGGGAGCCAACTCTAGGCTGATGGACTCCGCTAGCGGCGCCGGTCCTAAGAAGAAGAGGAAAGTGTGACTAGAGCTCGCTGATCAGCCTCGACTGTGCCTTCTAGTTGCCAGCCATCTGTTGTTTGCCCCTCCCCCGTGCCTTCCTTGACCCTGGAAGGTGCCACTCCCACTGTCCTTTCCTAATAAAATGAGGAAATTGCATCGCATTGTCTGAGTAGGTGTCATTCTATTCTGGGGGGTGGGGTGGGGCAGGACAGCAAGGGGGAGGATTGGGAAGACAATAGCAGGCATGCTGGGGAACAACGCCAAAGCAAAGAGTGAAACAGGGGGATCCTATCACAAAAGAATAAAGCAGCCTGAttggaaaacaaagagtggcgcttcttttctttcacattttctcagctcggcttctagcagaagccgtcctaggaaggagagggtttggagagttggggcgctttgccacgtcctttctaagagtgatggaggtttggtcctaccccaggtaatggggaacagagcaaaaggcagggacagaggacgggacctgggtgccatacacgggtgttaggcgccctccagcggtgtgtccacaaggaaggacagcgacagagacaggcagggagacagctagacagagagacagtgagaagcgagagagccgagatctaagacagacagactctggagtctctgggagagaacgatcgatcgctgatccagtgtctaagaggaaggaacggacctgacagcgtgtcccgtgcccggaggcactccgcctggtacaggacagcggcctgtagggggcccctcgcggagcggtgcctccgggcatccctggcccgcgccccctccccggcccctgccccagtcccaggcagcggattcccctccggggcgggcggtgctactctcgtcctccccgcccggctggcgtgcttcccggcccggccgagcacggtcccggccccgaggggggctgagctgggcgaaaacccgccctccagcgagctcatttccctaaaagggggggtggggtggggagtgggagggcggcgagaaaagaaagggagcgaccgagaggagggcgaggggacgccggagcccagggccggagagagccgggccgagcggggggcgggagacaggaaacgggacgggaagaggtggcctcggggggagagcgcctccccttcgccttcagcgctcccttccccgtcgccccgctcccgcgtccgggcgcgaccgccaccgccaccgccgcccgccagcatgcccggcgtggcccgcccgccgctgccgctgctgtcgctgccgctgctactgctgctgctgctgctcccgcgcgccggccggccgctggacttggccgactacacctacgacctgggcgaggaggacgccccggagctcctcaactacaaagacccttgcaaggcgggtgagcgacccccggctcccccagggcgcacggaagccgggcgtgggcaggctgggatcgaggctcccggccgggcaggggttggggttggggttgtagttggggttgggggaggacagtccagtgtgggaagctgggagctgcttggttggcaatgcagtgggggaacaaaagaccgagggagaaagggagggggaagtttgggggacttttaggactgaagttttgctgctgtttgtggaaactgctgcggctgccgttcgccttcacagaggaatcctggggaaacctccctcggtcccagcgaatcccgctggcaaggaggctttttctccaccgggaaactttactggccaaactgtgtgtgtgtgtgtgtgtgtgtgtgtgtgtgtgtgtgtgtccgtccgtccgtccgtccgtgtcccttctcccctatccctcctgctttgtggaatactggtgtttgcctgcttgtctgtttgccatagtgccgcacagcacatctgtctggtttgatgcccagaaagaaggcctgcagctccaaagggatagattttcccggcttctcagcccagtggaaggagaaagtttttcaggaggttctatggaatacttcttcataggcaatcctatgaatctagccagcagcaggagctctctgaacactcaagagacagctgtcaaagtcagagaaattggcggcaggggtgggggcttggggggcacagggggacttacctgtgcctttgtgtgccaggagagctttgccagagctcagttaggttagcaaatgagtctcacaaagaggtctcagatattctacatcagctctctcgccctacagacacctgaggctaattagtcaagtcagggcttctagtgtgtgtgtgtgtgtgtgtgtgtgtgtgtgtgtgtgtgtgtgtgtgtgtgatctttgttcatgaggactctcctgctgtgattcccttggagtgaccagatgaccagacacactcaggccattggcctaattgatgttcccaagcaggctgtctggaatacactggggacaagttatcacaacttttcctcgacttcccgcttctgacttcagcctgtctgcgaagagctagagccagggattgggacacaagaaatggacacaaagttttgggacaggcagctacaatcttgagcacagaggacctaatcagggactctccttgctccatccacccttaggtaggatagggaccattttacaggacaggacaaaccaaacccttcagtctttcctcttccatgcctggtatgctgtaggcaaaccaggacagtaaaagtctccatcacatcgtgcgtggtaggagggcaggaggaagaatgagcaaggcatgaaatctgaaggactcattagccaccaggaaggctgagcacagtctctgaacttaagttcctcattagagaactagctggtgggtaagggctaaatctgagaacatatataattcacttattattaacacacagcagacactatataaatgttcatttcctgtgctctgtgcctccagctcttgatcaatgtctttgagagggtgtggatttggattttagaaaaagctaacatctctctgaccctgtacggtgtctcatatccattcagtgatgttcgtttcttgatatgtctcagggctgggtttgatgttgtgggaggtgcagagagttgccagacagatctcggtccttaaagagatcacgttctaagcaagtgtccaagagcacactaatgttacacagcataattcaggaagattagggtccctcatcatctcttcgttcctggtgatggggagggcagagcaaagtggtttgaaggtgtgtgggaaggtttgtagagtggagattttttgtggaggcaGATCCTCTAGAGTCGAGCAGAACACGGAAGGAGACAATACCGGAAGGAACCCGCGCTATGACGGCAATAAAAAGACAGAATAAAACGCACGGGTGTTGGGTCGTTTGTTCATAAACGCGGGGTTCGGTCCCAGGGCTGGCACTCTGTCGATACCCCACCGAGACCCCATTGGGGCCAATACGCCCGCGTTTCTTCCTTTTCCCCACCCCACCCCCCAAGTTCGGGTGAAGGCCCAGGGCTCGCAGCCAACGTCGGGGCGGCAGGCCCTGCCATAGCCACGGGCCCCGTGGGTTAGGGACGGGGTCCCCCATGGGGAATGGTTTATGGTTCGTGGGGGTTATTATTTTGGGCGTTGCGTGGGGTCAGTCCACGACTGGACTGAGCAGACAGACCCATGGTTTTTGGATGGCCTGGGCATGGACCGCATGTACTGGCGCGACACGAACACCGGGCGTCTGTGGCTGCCAAACACCCCCGACCCCCAAAAACCACCGCGCGGATTTCTGGCGCCGCCGGACGAACTAAACCTGACTACGGCATCTCTGCCCCTTCTTCGCTGGTACGAGGAGCGCTTTTGTTTTGTATTGGTCACCACGGCCGAGTTTCCTCGACCGATGCCCTTGAGAGCCTTCAACCCAGTCAGCTCCTTCCGGTGGGCGCGGGGCATGACTATCGTCGCCGCACTTATGACTGTCTTCTTTATCATGCAACTCGTAGGACAGGTGCCGGCAGCGCTCTGGGTCATTTTCGGCGAGGACCGCTTTCGCTGGAGCGCGACGATGATCGGCCTGTCGCTTGCGGTATTCGGAATCTTGCACGCCCTCGCTCAAGCCTTCGTCACTGGTCCCGCCACCAAACGTTTCGGCGAGAAGCAGGCCATTATCGCCGGCATGGCGGCCGACGCGCTGGGCTACGTCTTGCTGGCGTTCGCGACGCGAGGCTGGATGGCCTTCCCCATTATGATTCTTCTCGCTTCCGGCGGCATCGGGATGCCCGCGTTGCAGGCCATGCTGTCCAGGCAGGTAGATGACGACCATCAGGGACAGCTTCAAGGATCGCTCGCGGCTCTTACCAGCCTAACTTCGATCACTGGACCGCTGATCGTCACGGCGATTTATGCCGCCTCGGCGAGCACATGGAACGGGTTGGCATGGATTGTAGGCGCCGCCCTATACCTTGTCTGCCTCCCCGCGTTGCGTCGCGGTGCATGGAGCCGGGCCACCTCGACCTGAATGGAAGCCGGCGGCACCTCGCTAACGGATTCACCACTCCAAGAATTGGAGCCAATCAATTCTTGCGGAGAACTGTGAATGCGCAAACCAACCCTTGGCAGAACATATCCATCGCGTCCGCCATCTCCAGCAGCCGCACGCGGCGCATCTCGGGCAGCGTTGGGTCCTGGCCACGGGTGCGCATGATCGTGCTCCTGTCGTTGAGGACCCGGCTAGGCTGGCGGGGTTGCCTTACTGGTTAGCAGAATGAATCACCGATACGCGAGCGAACGTGAAGCGACTGCTGCTGCAAAACGTCTGCGACCTGAGCAACAACATGAATGGTCTTCGGTTTCCGTGTTTCGTAAAGTCTGGAAACGCGGAAGTCAGCGCCCTGCACCATTATGTTCCGGATCTGCATCGCAGGATGCTGCTGGCTACCCTGTGGAACACCTACATCTGTATTAACGAAGCGCTGGCATTGACCCTGAGTGATTTTTCTCTGGTCCCGCCGCATCCATACCGCCAGTTGTTTACCCTCACAACGTTCCAGTAACCGGGCATGTTCATCATCAGTAACCCGTATCGTGAGCATCCTCTCTCGTTTCATCGGTATCATTACCCCCATGAACAGAAATCCCCCTTACACGGAGGCATCAGTGACCAAACAGGAAAAAACCGCCCTTAACATGGCCCGCTTTATCAGAAGCCAGACATTAACGCTTCTGGAGAAACTCAACGAGCTGGACGCGGATGAACAGGCAGACATCTGTGAATCGCTTCACGACCACGCTGATGAGCTTTACCGCAGCTGCCTCGCGCGTTTCGGTGATGACGGTGAAAACCTCTGACACATGCAGCTCCCGGAGACGGTCACAGCTTGTCTGTAAGCGGATGCCGGGAGCAGACAAGCCCGTCAGGGCGCGTCAGCGGGTGTTGGCGGGTGTCGGGGCGCAGCCATGACCCAGTCACGTAGCGATAGCGGAGTGTATACTGGCTTAACTATGCGGCATCAGAGCAGATTGTACTGAGAGTGCACCATATGCGGTGTGAAATACCGCACAGATGCGTAAGGAGAAAATACCGCATCAGGCGCTCTTCCGCTTCCTCGCTCACTGACTCGCTGCGCTCGGTCGTTCGGCTGCGGCGAGCGGTATCAGCTCACTCAAAGGCGGTAATACGGTTATCCACAGAATCAGGGGATAACGCAGGAAAGAACATGTGAGCAAAAGGCCAGCAAAAGGCCAGGAACCGTAAAAAGGCCGCGTTGCTGGCGTTTTTCCATAGGCTCCGCCCCCCTGACGAGCATCACAAAAATCGACGCTCAAGTCAGAGGTGGCGAAACCCGACAGGACTATAAAGATACCAGGCGTTTCCCCCTGGAAGCTCCCTCGTGCGCTCTCCTGTTCCGACCCTGCCGCTTACCGGATACCTGTCCGCCTTTCTCCCTTCGGGAAGCGTGGCGCTTTCTCATAGCTCACGCTGTAGGTATCTCAGTTCGGTGTAGGTCGTTCGCTCCAAGCTGGGCTGTGTGCACGAACCCCCCGTTCAGCCCGACCGCTGCGCCTTATCCGGTAACTATCGTCTTGAGTCCAACCCGGTAAGACACGACTTATCGCCACTGGCAGCAGCCACTGGTAACAGGATTAGCAGAGCGAGGTATGTAGGCGGTGCTACAGAGTTCTTGAAGTGGTGGCCTAACTACGGCTACACTAGAAGGACAGTATTTGGTATCTGCGCTCTGCTGAAGCCAGTTACCTTCGGAAAAAGAGTTGGTAGCTCTTGATCCGGCAAACAAACCACCGCTGGTAGCGGTGGTTTTTTTGTTTGCAAGCAGCAGATTACGCGCAGAAAAAAAGGATCTCAAGAAGATCCTTTGATCTTTTCTACGGGGTCTGACGCTCAGTGGAACGAAAACTCACGTTAAGGGATTTTGGTCATGAGATTATCAAAAAGGATCTTCACCTAGATCCTTTTAAATTAAAAATGAAGTTTTAAATCAATCTAAAGTATATATGAGTAAACTTGGTCTGACAGTTACCAATGCTTAATCAGTGAGGCACCTATCTCAGCGATCTGTCTATTTCGTTCATCCATAGTTGCCTGACTCCCCGTCGTGTAGATAACTACGATACGGGAGGGCTTACCATCTGGCCCCAGTGCTGCAATGATACCGCGAGACCCACGCTCACCGGCTCCAGATTTATCAGCAATAAACCAGCCAGCCGGAAGGGCCGAGCGCAGAAGTGGTCCTGCAACTTTATCCGCCTCCATCCAGTCTATTAATTGTTGCCGGGAAGCTAGAGTAAGTAGTTCGCCAGTTAATAGTTTGCGCAACGTTGTTGCCATTGCTGCAGGCATCGTGGTGTCACGCTCGTCGTTTGGTATGGCTTCATTCAGCTCCGGTTCCCAACGATCAAGGCGAGTTACATGATCCCCCATGTTGTGCAAAAAAGCGGTTAGCTCCTTCGGTCCTCCGATCGTTGTCAGAAGTAAGTTGGCCGCAGTGTTATCACTCATGGTTATGGCAGCACTGCATAATTCTCTTACTGTCATGCCATCCGTAAGATGCTTTTCTGTGACTGGTGAGTACTCAACCAAGTCATTCTGAGAATAGTGTATGCGGCGACCGAGTTGCTCTTGCCCGGCGTCAACACGGGATAATACCGCGCCACATAGCAGAACTTTAAAAGTGCTCATCATTGGAAAACGTTCTTCGGGGCGAAAACTCTCAAGGATCTTACCGCTGTTGAGATCCAGTTCGATGTAACCCACTCGTGCACCCAACTGATCTTCAGCATCTTTTACTTTCACCAGCGTTTCTGGGTGAGCAAAAACAGGAAGGCAAAATGCCGCAAAAAAGGGAATAAGGGCGACACGGAAATGTTGAATACTCATACTCTTCCTTTTTCAATATTATTGAAGCATTTATCAGGGTTATTGTCTCATGAGCGGATACATATTTGAATGTATTTAGAAAAATAAACAAATAGGGGTTCCGCGCACATTTCCCCGAAAAGTGCCACCTGACGTCTAAGAAACCATTATTATCATGACATTAACCTATAAAAATAGGCGTATCACGAGGCCCTTTCGTCTTCAAGAA
